# Organisational factors that facilitate research use in public health policy-making: a scoping review

**DOI:** 10.1186/s12961-019-0490-6

**Published:** 2019-11-21

**Authors:** Mette Winge Jakobsen, Leena Eklund Karlsson, Thomas Skovgaard, Arja R. Aro

**Affiliations:** 1 Unit for Health Promotion Research, University of Southern Denmark, Niels Bohrs Vej 9, DK-6700 Esbjerg, Denmark; 2 Department of Sports Science and Clinical Biomechanics, Research and Innovation Centre for Human Movement and Learning and Research Unit for Active Living, University of Southern Denmark, Campusvej 55, DK-5230 Odense M, Denmark

**Keywords:** Research utilisation, knowledge translation, evidence-based policy, health policy, scoping study, organisational research use, organisational research capacity, individual research capacity

## Abstract

**Background:**

Although important syntheses and theoretical works exist in relation to understanding the organisational factors that facilitate research use, these contributions differ in their scope and object of study as well as their theoretical underpinnings. Therefore, from an exploratory angle, it may be useful to map out the current literature on organisational factors of research use in public health policy-making when revisiting existing theories and frameworks to gain further theoretical insights.

**Methods:**

Herein, a scoping review technique and thematic content analysis were used to bring together findings from both synthesised and empirical studies of different types to map out the organisational factors that facilitate research use in public health policy-making.

**Results:**

A total of 14 reviews and 40 empirical studies were included in the analysis. These were thematically coded and the intra-organisational factors reported as enabling research use were examined. Five main categories of organisational factors that advance research use in policy organisations – (1) individual factors, (2) the management of research integration, (3) organisational systems and infrastructures of research use, (4) institutional structures and rules for policy-making, and (5) organisational characteristics – were derived as well as 18 subcategories and a total of 64 specific factors, where 27 factors were well supported by research.

**Conclusions:**

Using a scoping review methodology, the intra-organisational factors influencing research use in policy-making (including individual factors) were systematically mapped and the theories applied in this area of research were assessed. The review findings confirm the importance of an intra-organisational perspective when exploring research use, showing that many organisational factors are critical facilitators of research use but also that many factors and mechanisms are understudied. The synthesis shows a lack of studies on politicians and the need for more theoretically founded research. Despite increased efforts to update the existing evidential and theoretical basis of research use, we still need frameworks that combine different approaches and theories to help us grasp the complex organisational mechanisms that facilitate research use in policy settings.

## Background

The systematic use of research as an integrated part of health policy-making remains a challenge [1, 2]. An evidence-informed decision-making (EIDM) approach has been promoted to ensure better integration of research into public health policy-making, where policy-makers rely on sources of evidence other than research [3–6].

Systematic reviews of the factors that influence research use in health policy-making have paid increasing attention to the organisational factors that affect research use [7–14]. The SPIRIT Action Framework, developed by Redman et al. [15], characterises organisational research capacity as a key component in bridging the gap between research and policy as practise. Redman et al. stated that internal capacity includes ‘*the value placed on research by the organisation (as demonstrated through its support and requirements for research use) and by individual staff; the tools and systems the organisation has to support research engagement; and the skills and knowledge of staff*’ [15].

Although significant syntheses and theoretical works exist in relation to understanding the organisational factors that enable research use [12, 15–19], these contributions differ in their scope and object of study as well as in their theoretical approach. By taking an exploratory angle when reviewing existing studies on organisational factors of research use in policy organisations and by using a conceptual map of these factors, researchers will be able to revisit existing theories and frameworks to test their applicability to policy-making in comparison to the exploratory map and gain further insights into paths for future investigations. Therefore, we brought together findings from both synthesised and empirical studies to map out the organisational factors that facilitate research use in health policy-making.

### Policy organisations and research use

Building on Anderson [20], we define public health policies as actions performed by governmental bodies, including civil servants and elected or non-elected board members, that deal with healthcare commissioning, disease prevention and health promotion. In doing so, we need to conceptualise policy organisations as they ‘*provide meaning by which actors transact their work, formulate policy, and allocate resources*’ [21].

We define a policy organisation as a coordinated group of people with a shared, authorised purpose of developing public policies. Policy organisations are not only organised systems of policy officials (managers, professionals, technical and administrative staff, whose primary role is to support political boards) and board members (who have the ultimate decision-making authority), they are also comprised of their members, who are part of a broader political institutional framework composed of a “*collection of rules and organized practises, embedded in structures of meaning and resources that are relatively invariant in the face of turnover of individuals and relatively resilient to the idiosyncratic preferences and expectations of individuals and changing external circumstances*” [22]. In this way, individuals employed by a policy organisation are active members of a social network of people with shared goals and practises; they are also subjects of institutional policy rules that govern their work and which are integrated into their organisational culture [23].

### Aims

In our review of knowledge on organisational factors that facilitate research use in public health policy-making, we examined a range of different studies across disciplines, study designs (including literature reviews) and policy areas. Outcomes of interest are the organisational factors that the authors of the included papers have reported as facilitating research use in public health policy-making. The purpose of this review is to create add-on knowledge to existing frameworks for understanding research use in policy organisations and to identify possible research gaps.

## Method

We applied a scoping review method, as described by Arksey and O’Malley [24], to map out the research. This technique allows for a rigorous synthesis and mapping of an extensive, complex body of knowledge, providing the ability to extract findings exclusively related to organisational factors that enable research use. This approach is also useful for summarising findings from different study designs and theoretical backgrounds, permitting us to review the extent and range of the literature.

In line with the recommendations from Arksey and O’Malley, we excluded a quality assessment of the included studies [24]. Due to the scope and purpose of the review, we left out a consultation of preliminary results, which Arksey and O’Malley declared an optional stage.

### The scope of the review

This review focuses on the facilitating factors that support positive change mechanisms for integrating research within policy organisations to ensure EIDM. Our search strategy was not guided by a strict definition of research. Instead, we developed a comprehensive list of inclusion and exclusion criteria to capture all relevant studies on organisational factors that facilitate research use in policy organisations. Therefore, we did not distinguish between investigations conducted internally by policy officials or studies conducted by external academic institutions, nor did we distinguish between research evidence reported in peer-reviewed publications or evidence-based guidelines or verbally disseminated knowledge coming from such publications (e.g. by consulting with researchers).

There are many methods to appraise research use and only limited agreement exist among scholars on what characterises successful use of research. In our scoping review, we were not strict in our characterisation of research use; instead, we reported on the utilisation measure(s) applied in each study.

### Identifying relevant studies

**Fig. 1:**
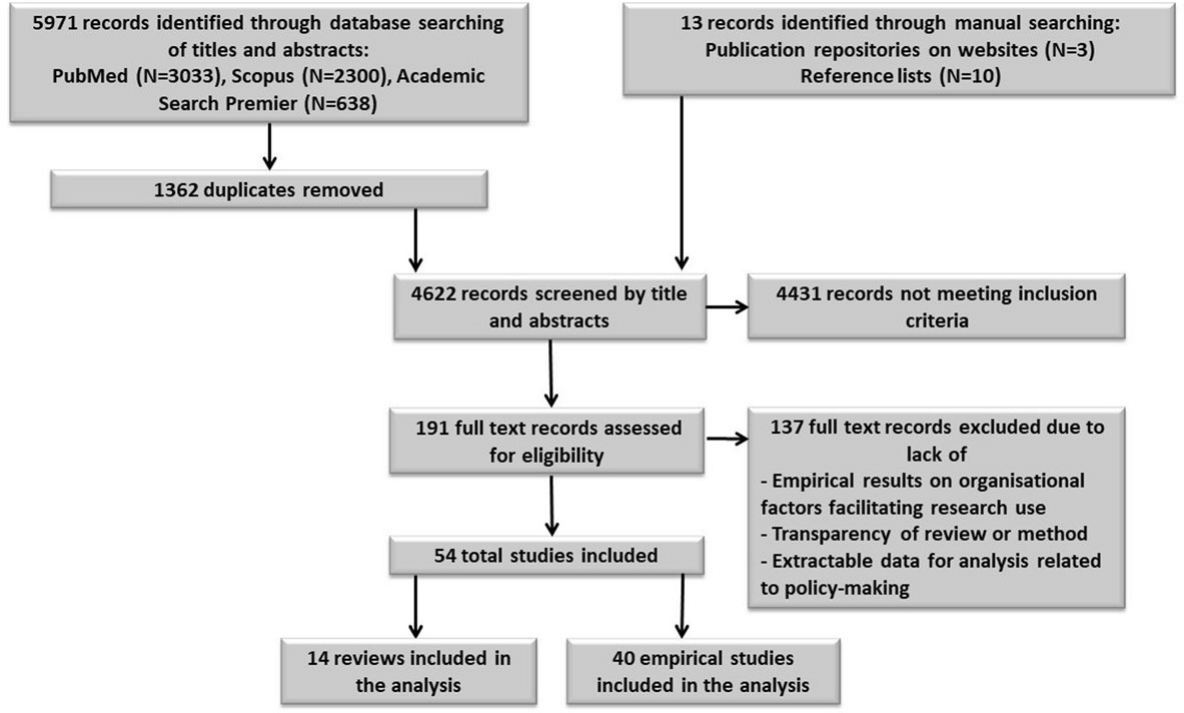
Review search strategy. Flow chart of the search strategy and the selection process for identifying studies reporting or synthesising empirical findings on organisational factors facilitating research use in public health policy-making

Between April and July 2017, we performed a combined search for relevant empirical studies published from 1970 to 2017 (July) in electronic databases, including PubMed, Academic Search Premier (cross-disciplinary) and Scopus (the social sciences). We also conducted a manual screening of document repositories on institutional websites known to have contributed to the knowledge base for enhancing evidence-informed/evidence-based public health policy-making, and we searched for important references already cited in the included studies. Figure 1 depicts our search strategy as well as the selection process.

#### Electronic databases

We initially performed a broad search on PubMed, specifically using the thesaurus Medical Subject Headings (MeSH), keywords and text words to include the most recent and pertinent publications. This database primarily includes publications in English. The aim of this search was to capture sources studying various types of organisations, which might be relevant in relation to research use and policy-making.

Thereafter, we performed four block searches using a wide range of search terms (Additional file 1), which we combined using the Boolean operators ‘AND’ or ‘OR’. We divided the block into the following focus areas: (1) population/setting, (2) intervention, (3) policy area and (4) theory development. The third focus area included health policies and public policies related to health. Where possible, we limited all searches to titles and abstracts. We did not look for keywords. Instead, we included many text words in each block search. The PubMed search revealed studies of practise-based organisations (such as hospital departments and mental healthcare facilities) that did not meet the inclusion criteria. We complemented our search using the Scopus and Academic Search Premiere databases, which are primarily social sciences oriented, but also cover many other disciplines such as humanistic research, economics, public health and the technical sciences. These two databases also contain publications other than those that are peer-reviewed. We modified the search strategy to exclude subject headings. We included studies of health services organisations if the abstract explicitly mentioned the word ‘policy’. We included ‘factors’ in the fourth block search to expand the focus on organisational factors of research use. We combined and merged the hits from these two databases into the PubMed publication repository in Endnote. The complete literature search strategy for all databases is available in Additional file 1.

#### Manual searches

Relevant websites and their publication repositories (both peer-reviewed and other publications) were screened based on the title of the publication and, if in doubt, the abstract. We included studies from the following two websites: (1) the Research Unit for Research Utilisation [25] and (2) Health Evidence™ [26] (funded by the National Collaborating Centre for Methods and Tools in Canada).

We screened reference lists of studies identified as pertinent from the database and website searches for key contributions to the review question. We identified 10 sources as being primary resources for other studies and highly relevant [8, 9, 27–34].

### Selection of studies

The primary researcher (MJ) screened all 4612 titles and abstracts through ongoing discussion with the second author (LEK) regarding the inclusion and exclusion criteria as well as the uncertainties related to some of the studies. Occasionally, a full text review was performed to refine the inclusion and exclusion criteria. By October 2017, 191 studies were found to be eligible for a full-text reading.

#### Inclusion and exclusion criteria

Table 1 presents the inclusion criteria. We developed and refined the exclusion criteria during the selection process. The eligibility of the grey literature and the manually searched literature were assessed using the same criteria used to determine the literature derived from the database search. The manually selected literature, comprising the guiding documents while developing the search strategy, was re-assessed to check their eligibility for inclusion.

**Table 1:** Inclusion and exclusion criteria

Inclusion criteria	
• English and Scandinavian language peer-reviewed and grey literature published between January 1970 and July 2017 reporting or summarising empirical findings on organisational factors of research use relevant for public policy-making. • We included studies focusing on organisational factors positively related to evidence-based or evidence-informed decision-making in public policy. • We included studies applying the diffusion of innovation theory by Rogers [35] if we identified the link between the ‘innovation’ and research use or evidence-based or evidence-informed decision-making from the title and abstract. • We included reviews of empirical findings and theories if the review method was clearly described.	
Exclusion criteria	
• Books, book chapters, book reviews, editorials, opinion articles, debate/discussion articles and comments on articles. • We excluded papers on research use if they did not focus on public policy-making within the policy organisation, for instance, implementation of screening programmes in community clinics, unless we found the word ‘policy’ in the title or abstract, and if we were able to extract the factors of interest. • We excluded studies that did not include policy-makers as study population, e.g. surveys of researchers’ perception of barriers and facilitators on research use in policy-making. • Study protocols were excluded unless they included empirical results from pilot testing. • We excluded papers if they only reported organisational barriers of research use, unless we deemed that the authors clearly stated that, by reversing one or more of the barriers, the factors would become facilitators. • We excluded papers focusing on research use through networking activities between policy-makers and external stakeholders, such as researchers, unless we were able to identify factors within the policy organisation clearly presented in the results as one of the main drivers of research use such as research capacity, governmental coordination or policy-makers’ preferences.	

We included one design paper as it was based on four preliminary studies, including two reviews [36]. The findings of the first (scoping) review are only available to us in this design paper, and the outcomes of the systematic review are also available in the form of a PhD thesis, but we were unable to retrieve this publication online.

### Charting and data extraction

We developed a data chart, which allowed us to extract information key to understanding the identified organisational factors. We recorded data on study characteristics (e.g. type and method, objectives and population as well as the outcome measures related to research use and theoretical foundation) and on the organisational context (e.g. organisational setting, policy area, level of policy-making and the country of study).

In order to pinpoint and thematically map out the organisational factors that facilitate research use, we applied thematic content analysis [37] to open codes of organisational factors identified in the study outcomes. Instead of using an existing coding frame, we developed our own, starting from the included reviews. Afterwards, we tested the coding frame while coding and thematically categorising a number of empirical studies (*n* = 17) [8–10, 27, 30, 33, 36, 38–47], which, through a text-search query in NVivo 11, were found to use the search term ‘organisational’ 10 or more times. We then open coded the remaining empirical studies and assigned these open codes the appropriate category from the existing coding frame. We used this step as a final validation of the categories and their definitions. The data extraction process enabled us to provide a frequency count of the identified organisational factors as well as their thematical and categorical attributes.

## Results

**Fig. 2:**
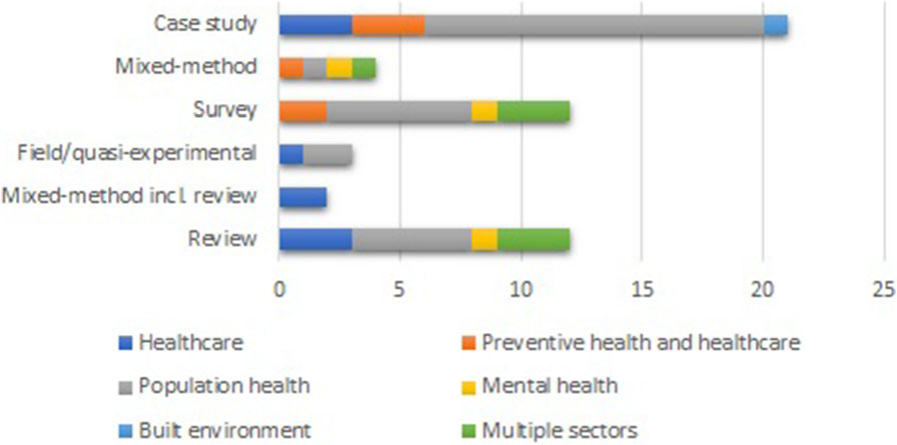
Study design characteristics and targeted policy area of included study. The graph shows the variation of the targeted policy areas for study design characteristic. Most noticeably is the large representation of studies conducted in the population health area, especially case studies, and the scares contribution of studies coming from the built environment and mental health areas

After the initial search, removing of duplicates and applying exclusion criteria, 54 publications remained eligible for inclusion. We classified the papers into 12 systematic or rigorous reviews, three field or quasi-experimental studies, 12 surveys, six mixed-method studies (where two of the studies included a systematic or rigorous literature review) and 21 case or naturalistic observation studies. We did not find any meta-analyses, randomised control trials or case-control trials. Figure 2 displays the targeted policy area of each included study type.

### Characteristics of the included studies

#### Reviews

We included 12 systematic or other types of literature reviews [11–14, 17, 29, 32, 48–52] and two mixed-method studies in our examination of reviews [16, 53]. These two mixed-method studies use empirical data to validate the findings of their own systematic review; thus, we have chosen to only consider these as reviews in our synthesis. A detailed summary of the included reviews, ordered by the number of organisational factors identified in each one, is presented in Additional file 2.

Organisational factors, in line with our definition, were seldom the primary object of investigation. The major contribution to this study stems from healthcare and population health (Fig. 2). Three reviews centre on multiple policy areas but report a large contribution from health policy and the level of policy-making in focus is quite equal across national/federal, state/regional and district/local levels.

#### Empirical studies

The empirical studies mainly consist of case studies and surveys from healthcare, preventive healthcare and population health policy. Most build on data from Australia, Canada and the USA. We only identified a few multi-country studies [39, 54–57], including two from African nations. In relation to policy, there is a very equal emphasis across levels, with the least focus on the state/regional level (national/federal (*n* = 22), state/regional (*n* = 17), district/local (*n* = 23)) (Additional file 3).

Civil servants are by far the most researched population group (Additional file 3). Politicians were only included in a few studies. However, many did not explicitly distinguish between elected and non-elected policy-makers.

The most commonly used measure of research use is policy-makers’ perceptions of barriers to, and facilitators of, research utilisation (Additional file 2). Only a few studies measured actual use [55, 58–61].

### Main approaches and theoretical foundations of the included studies

In our examination of reviews, most of the publications were explorative, rather than guided by theory. The main techniques of the included reviews stem from a rational action approach to policy-making, such as evidence-based/evidence-informed policy and knowledge translation (Additional file 2).

The included reviews are generally marked by the knowledge and research use approach, which revolves around the political components related to organisational research use [16, 29, 49]. Within this approach, Contandriopoulos et al. [12] provided a comprehensive understanding of the theoretical and empirical aspects of research use as knowledge in policy arenas (thus not only focusing on health). Contandriopoulos et al. reviewed both theory papers and empirical studies, which are combined in their synthesis, making facilitators with purely empirical bases unclear.

The largest theoretical contribution to the understanding of organisational factors, that enable research use in policy organisations, is the diffusion of innovation (DoI) theory by Rogers [35]. Several of the included reviews found this theory useful to grasp the individual and organisational mechanisms underlying the process, from the introduction of research evidence products (e.g. reports, systematic reviews, evidence-based guidelines) to the adoption of knowledge into organisational practises [14, 17, 48]. The concept of ‘absorptive capacity’ for new knowledge emerged from DOI literature; it is an organisation’s ability to be ‘*systematically able to identify, capture, interpret, share, reframe, and recodify new knowledge; to link it with its own knowledge base; and to put it to appropriate use*’ [14].

Regarding the understanding of organisational factors that enable research use, a couple of reviews applied the concept of organisational capacity or capabilities to use research [16, 53]. The SPIRIT Action Framework by Redman et al. [15] is the most recent theoretical contribution to the comprehension of organisational capacity to use research. This framework was based on an updated literature review by Moore et al. [17] and supplemented with interviews of policy-makers [16]. The framework builds on previous work from various disciplines [13, 14, 51, 62], with the aim of supporting increased efforts to develop evidence-informed health policy in Australia. Organisational capacity within the SPIRIT Action Framework is characterised as the extent to which an agency has the internal ability to respond to a catalyst or prompt for using research [15–17]. The organisational capacity of research use is influenced by the value the organisation places on research as well as through support and requirements for research use within the entity (e.g. support tools and systems for research engagement, skills development, and knowledge exchange and acquisition activities).

The theoretical backgrounds of the empirical studies were diverse and depended on the selected approach and interest(s) of the authors. For example, DoI, behavioural, and research impact and utilisation theories, in addition to a broad range of frameworks, have been used (Additional file 2). Below we present the theories that seem to have contributed the most to the field.

Nabyonga-Orem et al. [63] developed a middle range theory in 2012 in order to build understanding of the most likely circumstances for research use (i.e. knowledge translation) in policy-making by the ministries of health (MoH) in developing countries; the same team refined the theory in 2014 [59], stating the following: “*High-quality and contextualized evidence will be taken up in policies so as to lead to evidence-informed policies in instances where the MoH leads the KT* [knowledge translation] *process, the WHO and regional professional bodies play a role,* [there are] *partnerships for KT, and tools and required inputs are available to implement the evidence*” [59].

To support the development of knowledge exchange interventions, Contandriopoulos et al. [12] provide a framework for “*collective-level knowledge use*” that includes three dimensions of collective knowledge exchange systems: (1) “level of polarization” (from politics), where “*knowledge is influenced by its relevance, legitimacy, and accessibility*” (ibid, p. 460–461); (2) “*cost-sharing equilibrium*” (from economics), where the value for money of investing in knowledge exchange processes is constantly being assessed by knowledge producers, intermediaries and users; and (3) “*institutionalized channels of communication”* (from social structuring), where the “*social structures in a communication network influence the knowledge exchanged*” (ibid).

The included studies reveal that much of the evidence on research use does not contain an intra-organisational perspective. This was also recognised by Lomas and Brown, who proposed a new functional view of the role of research and the relationship between research use and health policy as well as a perspective from inside policy organisations whereby civil servants use research depending on the different functions that research plays in the policy-making process. They distinguished between the functional role of agenda-setting, policy development, and monitoring and modifying existing policies [64]. Here, the value of research by individuals and organisations is a key component to applying research for different purposes (i.e. functional roles). They stated that research is only taken into account for the following when it is valued: signalling what emerging or neglected areas may need to be on the agenda; verifying the issues claimed by interest groups to be worthy of inclusion on the policy agenda; as a source of validation for developing new policies; or as a learning process when using evaluations to monitor and modify existing policies. Lomas and Brown stressed the importance of valuing research within a learning culture [14]. This is in line with the cost-sharing equilibrium, which Contandriopoulos et al. [12] presented in their synthesis. When a cost-sharing equilibrium exists, research is more likely to be used.

The functional view of the role of research helps to provide an understanding of organisational culture and the kinds of ‘business problems’ that policy organisations face throughout the different phases of policy-making and how to best resolve them. Lomas and Brown [64] described a case of a state MoH with a learning culture that needed organisational and information tools to make better use of the advantages that research evidence could give civil servants, specifically when managing competing interests during policy agenda-setting, validating new policy recommendations and increasing the effectiveness of existing policies.

The DoI theory has also been used as the basis for empirical studies, and in this way contributed helpful understanding of the organisational factors that enable research use. This is especially the case for healthcare practise, where evidence-based guidelines and new technologies are usually the ‘innovations’ that can easily be defined as individual research products. In this review, two studies deployed the DoI theory to analyse the uptake of specific research products: clinical practice guidelines [42] and systematic reviews [27]. Dobbins et al. [27] argued that “*systematic reviews are an innovation because they represent a new approach for program planning and decision-making in public health*”. A third study used the DoI theory to develop the logic model of the knowledge translation intervention [36].

### Organisational factors that facilitate research use

We identified five broad domains of organisational factors, 18 subcategories and 64 specific organisational factors that enable research use (Table 2). The overall categories, subcategories and specific factors constitute the output of the thematic content analysis of the included reviews and empirical studies. This table provides, for each factor, the number of studies and their references reporting the factor so as to facilitate research use.

**Table 2:** Organisational factors of research use in policy-making identified in the included studies

Thematic overview of the organisational factors/Policy level and population studied by number of studies	Local	State/regional	National/federal	International	Civil servants	Politicians	Service managers and clinical/field staff	Researchers	Other external actors
INDIVIDUAL FACTORS									
External knowledge exchange linkages									
1. Brokering knowledge from different sectors and stakeholder groups (2 empirical studies [56, 60])	1	2	2		2			1	1
2. Informal, personal and trusting relationship with researchers (4 reviews [16, 29, 32, 53], 3 empirical studies [56, 60, 65])	5	7	6		8		2	3	4
3. Time spent on networking activities and acquiring research knowledge (1 review [48], 1 empirical study [41])	2		1		2				
Gender and age									
4. Being female (2 empirical studies [41, 66])	1	1	1		2				
5. Being younger or recent graduate (2 reviews [29, 32])	2	2	2		2		2	1	1
6. Seniority and having decision-making authority (1 review [48], 2 empirical studies [2, 27])	2	1	1		3				
Individual values, interests and beliefs									
7. Having a left-leaning political orientation (1 review [29])	1	1	1		1		1	1	1
8. Level of association and perceived relevance, credibility and objectivity of external research providers (4 reviews [12, 29, 32, 49], 4 empirical studies [8, 58, 60, 67])	5	9	7	2	9	2	2	4	5
9. Motivation, intention and expectations towards using research, including its perceived usefulness (7 reviews [12–14, 17, 29, 32, 48], 13 empirical studies [8, 9, 27, 30, 33, 41, 43, 47, 54, 56, 67–69])	13	10	13	1	19	2	4	3	6
10. Ownership of research results (1 review [51])	1	1	1		1		1	1	
11. Positive experiences with research translation and research use (1 review [29], 2 empirical studies [60, 69])	2	3	4		4		1	2	2
Position, status and role in the organisation									
12. Being a knowledge broker, champion or research gatekeeper (3 reviews [13, 14, 51], 6 empirical studies [10, 55, 58, 61, 70, 71])	5	4	5		9		4	5	2
13. Being an influential member of the organisation in promoting research (5 reviews [12–14, 49, 51], 2 empirical studies [10, 55])	5	5	6	2	6	2	2	4	3
14. Having a type of specialisation (1 review [48])	1				1				
15. Having decision-making authority (4 reviews [12, 13, 32, 48], 5 empirical studies [2, 41, 43, 66, 72])	8	5	8	1	11	1	1	2	3
Research awareness and integration skills									
16. Competencies and the ability to champion research use in a political setting (3 reviews [16, 17, 49], 4 empirical studies [45, 70, 73, 74])	4	4	4	1	7	1	2	1	3
17. Availability of internal experts with research knowledge in a particular policy area (3 reviews [14, 17, 32], 2 empirical studies [27, 73])	4	4	4		5		3		1
18. Having a high educational level (1 review [48], 6 empirical studies [2, 8, 9, 41, 66, 72])	3	4	4		7				1
19. Having a low educational level (1 empirical study [75])	1				1				
20. Having research experience and skills (4 reviews [16, 17, 29, 52], 7 empirical studies [33, 41, 45, 66, 67, 75, 76])	7	7	5		11		3	2	4
21. Skills in seeking, appraising and interpreting systematic reviews and adapting to contextual needs (5 reviews [14, 16, 17, 32, 53], 9 empirical studies [38, 39, 42, 45, 47, 54, 56, 68, 73])	8	4	9		13	1	3	1	5
MANAGEMENT OF RESEARCH INTEGRATION									
Performance management									
22. Availability and organisation of internal staff, which coordinate and respond to specific demands for research to inform a policy (1 review [53], 8 empirical studies [27, 30, 61, 64, 67, 74, 76, 77])	7	6	5		12		3	2	5
23. Continuity and stability of employment for high level leadership and staff (2 reviews [29, 48], 2 empirical studies [30, 45])	4	1	1		4		1	1	1
24. Development of shared positions or exchange programmes with university (1 review [53], 4 empirical studies [60, 64, 65, 69])	2	4	5		6			2	3
25. Internal capacity-building (9 reviews [11, 13, 14, 16, 17, 32, 51–53], 17 empirical studies [27, 30, 33, 36, 38, 42, 45, 46, 54, 56, 59, 60, 64, 67, 69, 71, 73])	18	12	16	1	26		6	6	8
26. Research integration skills, which form an essential part of recruitment policy and the performance management system (2 reviews [16, 53], 6 empirical studies [30, 33, 42, 45, 61, 72])	5	3	2		7		1		2
Strategic commitment towards research use									
27. Clear strategic vision for, and the systematic incorporation of, research use within existing systems and practises (3 reviews [13, 14, 51], 7 empirical studies [10, 30, 44–46, 64, 77])	7	4	5		10		2	3	2
28. Efforts to create an organisational culture favouring research use (6 reviews [11–14, 17, 51], 3 empirical studies [30, 33, 73])	8	6	7	2	9	1	3	4	2
29. Provision of sufficient time and resources to acquire research, make decisions and engage with research activities (7 reviews [12–14, 17, 29, 32, 51], 10 empirical studies [8, 30, 33, 36, 41, 42, 45, 56, 59, 67])	14	8	11	1	16	1	6	5	6
30. Support by senior managers (3 reviews [14, 29, 51], 8 empirical studies [10, 36, 38, 42, 45, 58, 64, 75])	8	6	4		11		3	3	2
ORGANISATIONAL SYSTEM AND INFRASTRUCTURE FOR RESEARCH USE									
Access to research									
31. Access to online or in-house databases and repositories of research (5 reviews [12, 17, 29, 32, 53], 7 empirical studies [8, 10, 27, 36, 46, 60, 64])	7	9	6	1	12	1	3	2	4
32. Personal access to a researcher, research consultant or internal expert (4 reviews [29, 48, 53], 3 empirical studies [36, 60, 73])	4	3	5		7		2	1	2
33. Provision of library services or support by an information specialist (3 reviews [29, 32, 53], 4 empirical studies [10, 71, 73])	3	3	4		6		2	1	2
34. Availability of tailored, disseminated research findings to policy-makers (4 reviews [12, 17, 29, 53], 4 empirical studies [43, 64, 70, 77])	4	5	7	1	8	1	3	4	7
35. Technical support to access research findings (1 empirical study [39])	1		1		1	1		1	1
Inter-organisational communication and collaboration									
36. External partnerships and communication channels (5 reviews [12, 29, 48, 52, 53], 9 empirical studies [39, 44, 56, 58–60, 76–78])	9	8	15	1	14	2	3	8	10
37. Government and academia collaborative research (1 review [53], 5 empirical studies [56, 58, 60, 74, 77])	2	4	5		7			3	4
Intra-organisational communication, learning networks and collaboration teams									
38. Clear messages and good internal networks among leaders across departments (1 review [14], 2 empirical studies [45, 73])	2	1	2		3		1		
39. Intra-organisational communication and learning networks (3 reviews [14, 48, 52], 7 empirical studies [8, 30, 44, 59, 70, 73, 74])	7	4	6		10		4	3	4
40. Multidisciplinary and multiagency teams (3 review [14, 50, 52], 1 empirical study [70])	3	3	4		4		4	3	3
Knowledge management systems and methods for internal research generation									
41. Availability of a comprehensive knowledge management system for research use (1 review [53], 1 empirical study [30])	1		1		2				1
42. Data collection systems for research, monitoring and evaluation (2 review [14, 52], 2 empirical studies [10, 59, 79])	3	4	3		5		3	2	2
43. Methods for collecting and generating research to inform policy (1 review [16], 1 empirical study [76])		1			1				
INSTITUTIONAL STRUCTURES AND RULES FOR POLICY-MAKING									
Political environment									
44. Establishing platforms for engaging all stakeholders across sectors in policy discussions and where research evidence is discussed (2 reviews [52, 53], 2 empirical studies [59, 60])	2	2	4		4		2	2	3
45. Funding and commissioning of research (5 reviews [14, 16, 29, 32, 53], 9 empirical studies [30, 58–61, 64, 69, 73, 76])	5	7	7		13		4	3	3
46. Open and transparent policy-making process that creates opportunities for public input (1 review [48], 4 empirical studies [46, 59, 65, 76])	4	2	2		5		1	2	2
47. Political support and procedures for using research for policy-making (4 reviews [12, 16, 29, 53])	2	3	3	1	4	1	1	2	3
Implicit rules and preferences on how to make policy									
48. High value placed on questioning, experimentation and risk taking as part of the organisation’s culture (1 review [14], 3 empirical studies [10, 30, 74])	3	3	1		4		1		
49. High value placed on rationality, professionalism, speciality, measurement, evaluation and quality improvement as part of the organisation’s culture (1 review [14], 6 empirical studies [10, 45, 60, 61, 70, 76])	3	4	3		7		3	1	1
50. Shared importance and high value of research in policy-making as part of the organisation’s culture (5 reviews [13, 17, 32, 48, 51], 7 empirical studies [8, 9, 27, 58, 64, 73, 76])	6	8	6		11		3	3	1
ORGANISATIONAL CHARACTERISTICS									
Function of the organisation									
51. Being a healthcare organisation (1 empirical study [33])	1	1			1				
52. Being a statutory body that has to stand up to legal scrutiny (1 empirical study [61])	1				1		1		
53. Being an organisation with high functional differentiation (number of divisions or departments within the organisation) (1 empirical study [47])			1		1				
54. Being an organisation whose primary task focuses on policy and programme development (1 review [48])	1				1				
Size and complexity of the organisation									
55. Being a medium- or large-sized organisation and unit (1 review [48], 3 empirical studies [9, 33, 41])	3	2	2		4				
56. Being an organisation that provides a large number of distinct services (1 review [48])	1				1				
Policy area									
57. Working in a disease prevention policy area (1 empirical study [57])	1	1	1		1		1		1
58. Working in a policy area where political conflicts are low (1 empirical study [57])	1	1	1		1		1		1
59. Working in a policy area with a pathogenic focus (1 empirical study [57])	1	1	1		1		1		1
60. Working in a technical policy area (1 empirical study [59])	1		1		1		1	1	1
61. Working in an education or social policy area (2 empirical studies [9, 66])		2	1		2				
Level of policy-making									
62. Being a national level organisation (1 empirical study [57])	1	1	1		1		1		1
63. Being a provincial level organisation (1 empirical study [9])		1	1		1				
Location									
64. Being in an urban area (1 review [48])	1				1				

**Fig. 3:**
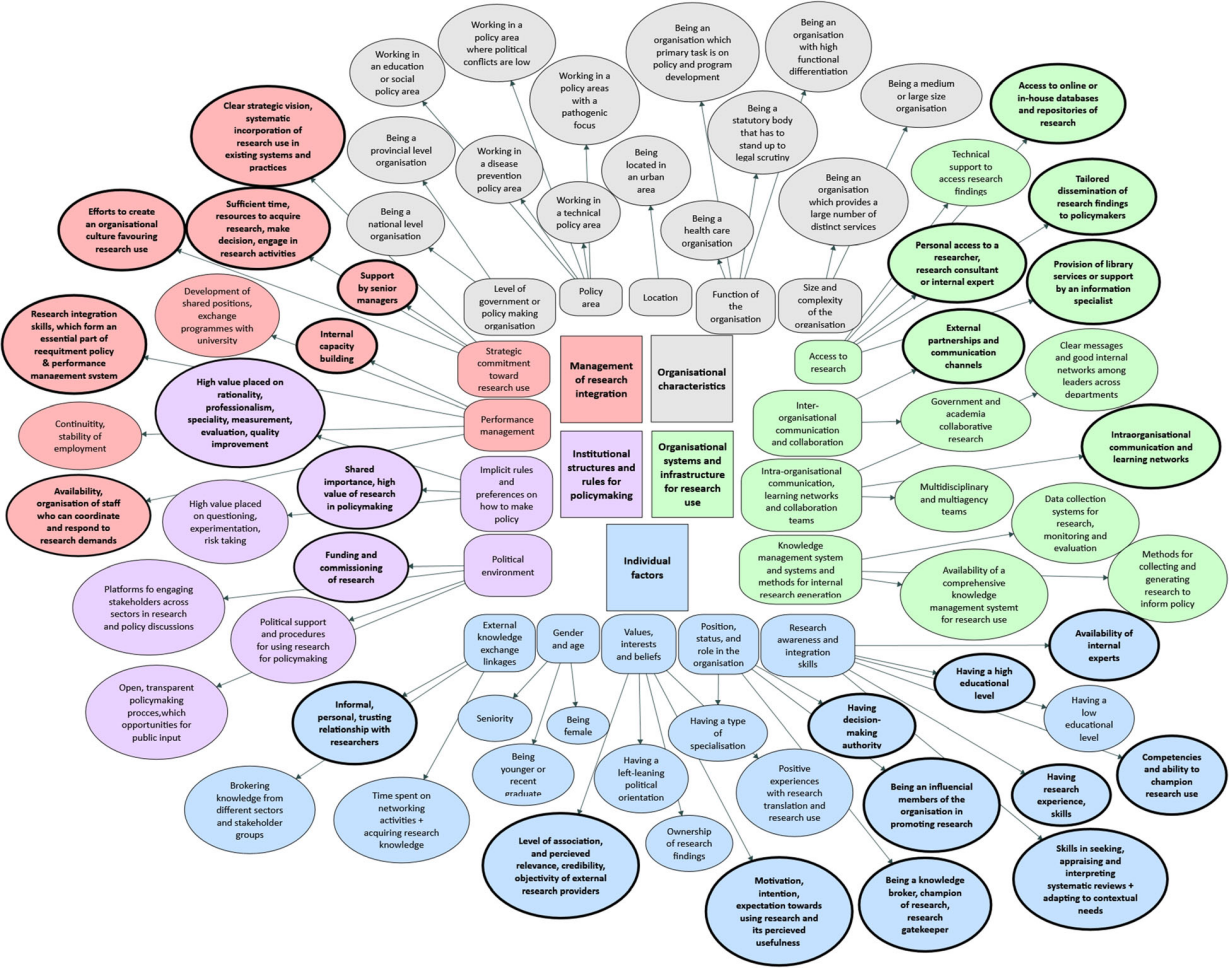
Concept map of organisational factors that facilitate research use including highly supported factors. The overall categories are displayed as squares, subcategories as squares with soft edges, and the detailed factors are displayed as circles. The sizes of the circles are without importance. The circles with bold text and thick boundaries present the factors, which are supported by seven or more studies including at least one review. The figure shows that 27 out of 64 identified factors are highly supported by research, primarily within the overall categories of individual factors, management of research integration and organisational systems and infrastructure for research use

Figure 3 displays the concept map of the organisational factors. The factors highlighted with a thick border are supported by seven or more studies, including at least one review. Additional file 3 summarises the organisational factors identified in each study (as presented in Table 2) as well as the policy level and study population of each included investigation.

#### Individual factors

This general category of factors has the largest support from research (44 investigations). Studies from all levels of policy-making feed into this category, where civil servants comprise the largest population group examined. The facilitating factor primarily supported by research is ‘motivation, intention and expectations towards using research, including its perceived usefulness’ (20 studies); it falls under the subcategory of ‘individual values, interests and beliefs’. The synthesis of findings encompassed by this factor show that the motivation to seek out and use research is driven by an individual’s opinions, preferences and interests as well as to what extent research is valued. This factor includes the perceived usefulness and impact of research as a means of improving policies and achieving policy goals.

Another closely related and empirically supported facilitating factor is the ‘level of association and perceived relevance, credibility and objectivity of external research providers’ (8 studies); it is also part of the ‘individual values, interests and beliefs’ subcategory. This factor clearly shows the importance of policy-makers to be able to employ research in order to realise policies [80]. In order to make use of research for political purposes, the research evidence has to meet the same criteria of judgement as other kinds of evidence, meaning that policy-makers should have confidence in review authors [32], placing importance on the reputation, professional credibility and legitimacy of the research providers (both individuals and their affiliated institutions) [49, 60]. Moreover, policy-makers judge the interests of the research providers when weighing the evidence [29].

Our review synthesis demonstrates that all factors except one – within the subcategory of ‘research awareness and integration skills’ – are well supported by studies (Fig. 3). The following two factors had the highest support of research: ‘skills in seeking, appraising and interpreting systematic reviews and adapting to contextual needs’ (14 studies) and ‘research experience and skills’ (11 studies). This means that skills and competences in both the generation and integration of research facilitate its use. Having a high educational level is supported by six empirical studies and one review (Table 2). One empirical study illustrates inconsistent results regarding the cause–effect relationship between high educational background and high research use, reporting on civil servants with low educational backgrounds as high users of research [75] (Factor #19, Table 2). Here, it might be relevant to assess the quality of contradictory studies and remain open to potentially underlying mechanisms connected to research use, which may be hidden under the education factor.

We identified having the ‘competences and being able to champion research use in a political setting’ as a pertinent facilitating factor of research use (7 studies); it entails the ability to champion research among those who are likely to be influential in getting research into policy, both internally and externally [17, 74]. It is vital to research integration that policy-makers be able to make decisions based on the best available research in a policy area filled with contingency and surrounding influence [74], financial constraints [45], time pressure [73] and using politically palatable arguments to gain political support for policy actions that are informed by research evidence [16, 49].

In the subcategory of external knowledge exchange linkages, the most supported factor is ‘informal, personal and trusting relationships with researchers’ (Fig. 3). This factor is related to the theoretical concept of social structuring, which both Contandriopoulos et al. [12] and Walter, Nutley and Davies [51] reported on. Interpersonal trust encourages communication and repeated communication raises trust. This means that communication must be initiated among research users and producers in order to build trust; these relationships are enhanced by ongoing communication. A supporting factor is the ‘time spent on networking activities and acquiring external research knowledge’.

Several findings are related to the subcategory of ‘position, status and role in the organisation’, which are understudied. Furthering the comprehension of personal characteristics that facilitate research use might be useful for strategic performance management that supports EIDM. It is also critical to grasp the personal implications of being a member of an organisation that advocates for EIDM (e.g. an individual’s perceived pressure whereby they feel the need to be an expert on many issues to make evidence-based/evidence-informed decisions) [40].

Two studies showed being female as a facilitating factor, since findings indicate that females use research more often than males [41, 66]. However, the same studies argue that this factor might not be feasible or it that it is not ethical to place too much emphasis on this. Another factor that might not be ethical to highlight is the potential association between having a left-leaning political orientation and research use; we identified this factor in a review [29].

Conflicting results have been found in relation to age and seniority, where seniority [2, 27, 48] and being younger or a recent graduate [29, 32] have both been identified as enabling research use. In the survey by Newman [66], age and level of work experience were not significantly associated with research use. Newman suggested that age does not heavily influence research use, but there is a far more complex explanation.

We found that the positive relationship between a decision-making authority and research use was more visible in the factor of ‘having decision-making authority’ under the subcategory of ‘position, status and role in the organisation’. Here, we identified research users as members in an organisation that hold a managerial or senior position whereby one is allowed to intervene in the practises, rules and functioning of organisational, political or social systems [12]. Furthermore, having a high professional role in an organisation favours the use of research [41].

We identified ‘being an influential member of the organisation in promoting research’ as a facilitating factor that is highly supported by research (Table 2). Having visionary staff in pivotal positions is relevant for a strong receptive capacity for research [14]. Having influential members of the organisation (both professionally, such as policy entrepreneurs, and socially, such as opinion leaders) is key to enabling research use. Another factor closely interconnected with the factor above is ‘being a knowledge broker, champion or gatekeeper of research’; this covers the availability of ordinary members who support influential members, who in turn are the main drivers of research use. Being the knowledge brokers and gatekeepers of research, these individuals validate the research quality and political applicability before using it or transferring knowledge to the rest of the organisation. This factor is directly linked to ‘competencies and the ability to champion research’. When analysing the differences of individual characteristics between research users and non-users among Australian government officials, Newman concluded that government academic research users tend to have much in common with academics that have previous experience in the university or non-profit sectors [66]. He stressed the importance of identifying individuals within policy organisations who can act as knowledge brokers [66].

#### Management of research integration

We mapped the management of research integration (such as performance management and strategic commitment toward research use) as the second most empirically supported overall category of factors. Forty-one studies have contributed to this general category, where most of the factors have emerged by studying civil servants at all policy-making levels.

As part of the ‘performance management’ subcategory, nine studies (Table 2) highlight the ‘availability and organisation of internal staff, which coordinate and respond to specific demands for research to inform a policy’. This factor includes the availability of internal staff/specialists who conduct literature reviews that are used to inform decision-making [30], and who have the analytical skills needed to evaluate unpublished sources of information such as agency administrative data [76].

For commissioning organisations, Wye et al. [61] revealed the importance of conducting one’s own evaluation studies; Lomas and Brown [64] support this. In the case of the selection and updating of Mali’s National Essential Medicines List, Albert et al. [67] stated that “*having a specific person or group of persons delegated to search and compile relevant research findings for the policy question at hand was perceived to be extremely helpful*”.

Having internal research units was reported as facilitating research use [27, 64, 74, 77]. For instance, Elliott and Popay reported that having a research resource centre enables cross-sector communication in relation to needs assessments and health services contracting [74]. Mwendera et al. [77] underscored the usefulness of a policy development unit to coordinate and implement evidence-informed policies.

Nine out of 14 reviews and 15 out of 40 empirical studies (Table 2) reported ‘internal capacity-building’ as a facilitating factor, making this the most studied factor of those identified in this review. In their survey of public health decision-makers in public health units in Ontario, Dobbins et al. [27] found that ongoing training in critical appraisal was the second strongest predictor of the use of systematic reviews for programme justification. Training sessions, which target both policy/project officers and senior managers, have been stressed as key to improving the confidence and expertise of policy-makers in all positions regarding research use in policy-making [36, 53]. Furthermore, better access to staff training has been demonstrated as a feature among local health departments, which have a high capacity to effectively implement and maintain evidence-based public health [45]. The reported content of the training and capacity-building mostly included literature search techniques, critical appraisal, local application, and monitoring and evaluation. Additionally, we identified shadowing sessions by librarians, mentoring and supervision as useful for capacity-building [42, 51, 71]. Formal methods for accessing research [16] as well as the availability of tools for searching, collating, synthesising, reporting and applying research [36, 46, 53, 59] were found to be important for supporting policy-makers during their capacity-building efforts.

Another facilitating factor included in this subcategory, that is highly supported by research, is ‘research integration skills, which form an essential part of recruitment policy and the performance management system’. This factor includes the incorporation of research use capacity and research skills into position descriptions [42, 53] as well as performance management systems for senior policy-makers [53]. Having people in the organisation who are paid to do research [33], and strategies in place to provide staff with training [16], influence individual skills and capabilities to integrate research. Developing performance objectives related to research in annual employment reviews was mentioned by Peirson et al. [30] as a “*means of providing regular and mutually accountable opportunities for staff and supervisors to identify EIDM learning needs, develop training plans, monitor progress, and assess performance*”. Giving staff opportunities for growth and involving them at all levels of decision-making was highlighted by Tabak et al. [45] and having incentive systems (like professional rewards for using and conducting research) was reported by Oh [72] as impacting the search for external research by civil servants.

All factors within the subcategory of ‘strategic commitment toward research use’ are highly supported by research (Fig. 3). The factor ‘provision of sufficient time and resources to acquire research, make decisions and engage with research activities’ was stated by 17 studies. Here, a wide range of resources have been reported on such as the availability of funding for research activities (e.g. flexibility of funding) [45] and the availability of slack resources [14]. Furthermore, emphasis was placed on the investment of significant time, efforts and funds to build internal library infrastructure and expertise [30] as well as investment in systems and processes that aid research use [17]. Resources for staff and personnel [29] as well as knowledge translation efforts [41] and technology [13, 14], were also mentioned.

Studies on research use in low-resource countries show a high influence by global institutions and international donor agencies, which greatly impact the perceived importance of research use in health policy-making [55, 77]. Government resources allocated for research have been identified as a critical organisational factor. However, such funding efforts may not be realistic in low-resource countries. In one study, even if there was no government funding available (e.g. in the case of malaria policy in Malawi), it was still possible for external research funding to support policy-makers’ needs by developing the National Health Research Agenda [77].

The importance of having a ‘clear strategic vision for, and the systematic incorporation of, research use within existing systems and practises’ was reported by 10 studies. This factor includes a clear, long-term strategy to increase research use [14, 30], whereby Peirson et al. [30] suggested a 10-year period. Other findings highlight the significance of systematically integrating evidence-based decision-making (EBDM) into strategic plans [45] as well as transparent roles and responsibilities for EIDM [13, 44, 46]. The development of research priorities for organisations was also mentioned such as having established committees, identifying research priorities for the organisation [77] and annually eliciting upcoming evidence priorities from staff [64]. The creation of a managerial position for workforce development and the priority of libraries in local public health units in Ontario were reported as facilitating research use [30]. Finally, systematising research use procedures across departments and within existing organisational systems is vital for enabling research use [10, 46, 51].

‘Support by senior managers’ was identified as an important factor by 11 studies. Walter, Nutley and Davies [51] stated that “*strong and visible leadership, at both* [the] *management and project levels, can help provide motivation, authority and organisational integration*”. Several studies mentioned leadership and managerial will and support as catalysing research, but without further clarification. Lomas and Brown [64] revealed the central importance of having champions at a senior level able to sustain the journey. Hardy et al. [38] reported that encouraging EBDM by leaders through positive feedback and follow-up on staff suggestions to improve practise and support enabled the implementation of EBDM at a local health department in Colorado. The development of a Senior Officers Group, with responsibility for overseeing strategies for carrying out research, was also mentioned as ensuring a positive outcome of research use [58].

The final factor included in this subcategory is ‘efforts to create an organisational culture favouring research use’. Nine studies contributed to this factor, which is complex, as efforts to change organisational culture are already embedded in other identified factors. However, during coding, there were codes referring explicitly to the significance of transforming an organisation’s culture to become one that promotes research use [11, 17, 33] (e.g. through institutional incentive schemes) [12]. By creating an organisational learning culture, the absorptive capacity of research and innovation is enabled [13, 14, 73]. Mitton et al. [13] reported on readiness for change as an organisational facilitator, without further clarification. Many of these cultural concepts are multi-factorial, and hence difficult to decompose into a more exploratory summary of factors, especially the codes included in this final one. Thus, we have refrained from doing so.

#### Organisational systems and infrastructures for research use

Thirty-two studies contributed to the overall category of ‘organisational systems and infrastructure of research use’, which includes factors related to the organisational systems and infrastructure in place for accessing and managing knowledge as well as for exchanging and discussing research knowledge within the organisation and with research producers. Factors under this overall category come from studying a wide range of policy levels and a broad swath of population groups, both inside and outside policy organisations (e.g. researchers, clinicians and employees of non-governmental organisations). This is very much due to the external linkages identified within this overall category.

Within the subcategory ‘access to research’, all factors except for one are highly supported by research (Fig. 3). Several of the factors in this subcategory are heavily dependent on external influences (e.g. dissemination activities from research providers, or external knowledge brokers and the provision of external library services).

The most empirically supported factor in the ‘access to research’ subcategory is ‘access to online or in-house databases and repositories of research’ (20 studies); this includes the permission to use, and the availability of, research via full-text access to online databases, mostly external findings (including to online database searches) [27, 32, 36, 53], or via internal repositories of research with both internal and external results. Subscription to all or the most relevant research journals [53] and the availability of systematic reviews [32] are contained in this factor.

Another factor is the ‘provision of library services or support by an information specialist’ (7 studies). Having access to library services, either internal or external, or an information specialist was reported by three reviews [29, 32, 53] and four empirical studies [10, 30, 71, 73] for the efficient retrieval of pertinent sources of information. However, the research does not adequately clarify which strategy is most effective. Twose et al. [71] found that access to highly skilled external library services and personal assistance by a librarian were useful for county officials working in two public health departments in the United States. However, this access was supported by ongoing training and shadowing sessions by the librarians, where peaks of use coincided with training and shadowing events. To accommodate the high needs of research by low research users, Twose et al. [71] proposed establishing “*power users*” – who might be already existing high users – in departments to assist with research integration, despite time restrictions faced by officials and the complex questions guiding literature searches. They also stress the need for a more seamless and broad-based model for accessing information versus the patchwork of services currently available [71].

The ‘availability of tailored, disseminated research findings to policy-makers’ was identified as one of the highly research-supported factors within the ‘access to research’ subcategory (8 studies). This factor encompasses active dissemination to policy-makers (e.g. by organisational leaders) [53] through the circulation of policy-relevant research findings and reports [53, 64] or through tailored, policy-friendly summaries [12, 17, 43, 53]. Regarding the frequency of dissemination, More et al. [17] mentioned the positive effect of an intervention that combined access to an online research repository with tailored weekly target messages for 7 weeks. Alternatively, Lomas and Brown [64] examined a quarterly newsletter combining government-funded and peer-reviewed research to support the functional demands of civil servants during their ‘pull’ of sources during short periods of new policy development.

In light of the findings by Oh [72], increased access to external sources of research indicates that civil servants are even more prone to looking for external sources if this information is given to them, if the research helps them to solve their daily tasks and if organisational incentives for external research exist. An increased use of external sources is mediated through greater efforts to spread information, or by influencing the needs of the research via decision-makers and increased collaboration efforts [27, 60, 72].

The final, most highly research-supported factor in the ‘access to research’ subcategory is ‘personal access to a researcher, research consultant or internal expert’. This factor might be closely linked to social structuring theory, according to which trust in the research provider affects the individual’s perceptions of, and relevance to, the research. Personal access to research providers – either from colleagues in a brokering position or from external researchers or a research consultant – supports an intuitive approach by policy-makers to acquire information. One review reported on the storing of tacit, un-codifiable knowledge within the organisation as a facilitating factor [14]. This factor is associated with DoI theory in healthcare practise, leaving its pertinence to policy-making unclear. However, personal access to people with specialised knowledge of research in a particular area [9, 73] or in acquiring research [53] could be connected to the organisation’s existing knowledge and skills base; hence, the storing of tacit knowledge is performed by selected members/power users in the organisation [14].

Studies on the effect of knowledge brokers as a knowledge translation strategy have mainly focused on external knowledge brokers (e.g. from research institutions) [13, 17, 49, 81]. These studies do not sufficiently describe who the internal knowledge brokers are.

Only the factor ‘external partnerships and communication channels’ was identified as being highly supported by research under the subcategory ‘inter-organisational communication and collaboration’ (14 studies). This factor includes participation in fora and conferences where research findings are presented [44, 53, 77] as well as fora and platforms where evidence is discussed and decisions are made in relation to research use for specific policy problems [39, 58, 59, 77]. Organised networking and partnership activities foster the development of interpersonal trust between policy-makers and researchers. Based on individual-level theories of human behaviour, Contandriopoulos et al. [12] argued that “*interpersonal trust facilitates and encourages communication*” and that “*repeated communications create trust*”. The importance of long-term, trust-based relationships for research was also highlighted by two studies [56, 59]. Van der Arend [60] examined reports from policy officials on how their participation in many different types of linkage relationships supported them in accessing, translating, commissioning and co-producing research. Nabyonga-Orem et al. [59] explored a positive case of research use under the strong leadership of Uganda’s MoH regarding knowledge synthesis and the application process during a change in malaria treatment policy.

Under the subcategory ‘intra-organisational communication, learning networks and collaboration teams’, only one factor (‘intra-organisational communication and learning networks’) was highly supported by research. In this factor, peer-to-peer networking [52] – also referred to as horizontal networks [14] – has been viewed as useful in creating shared meanings and values related to research use, whereby sharing research becomes part of the organisation’s social knowledge [14]. Nutley, Walter and Bland [70] revealed that developing mechanisms to bring analytical government staff together with their policy counterparts in a case of drug misuse facilitated research use. They highlighted policy fora as a useful way of bringing analysts and officials together on a regular basis and also suggested co-locating analysts and officials as a potential option. Peirson et al. [30] described the creation of tighter internal webs through established learning networks centred around capacity-building activities such as clubs for critical appraisal, reference managing or method application [30].

Having semi-autonomous ‘multi-disciplinary and multi-agency teams’ was reported by three reviews [14, 50, 52] and one empirical study [70] as enabling research use by balancing input from specialists and generalists, respectively. This factor is derived from two reviews based on DoI theory [14, 50]. We did not find any support for this factor instead of innovation adoption in the empirical studies, perhaps because it is still an understudied area in policy-making.

None of the factors under the subcategory ‘knowledge management systems, and systems and methods for internal research generation’ were highly supported by research. The ‘availability of a comprehensive knowledge management system for research use’ has been suggested, albeit with insufficient evidence, as an effective way of aiding EIDM [53]. Such a system should contain easy, accessible internal and external data, previous review search strategies and results (mentioned earlier by repositories of research findings), research integration tools and available systems for searching, collating and applying research, templates and manuals as well as all materials for documentation and auditing [30].

#### Institutional structures and rules for policy-making

Thirty-three studies on all policy-making levels have contributed to the general category of ‘institutional structures and rules for policy-making’. This category contains the explicit and implicit institutional rules and procedures that enable research to be integrated into policy-making and used in a rational, democratic manner. The factors in this overall category stem from studies on civil servants and external policy actors.

Under the subcategory ‘political environment’, the only factor highly supported by research is ‘funding and commissioning of research’. This includes the statutory commitment to fund, commission and use commissioned studies and activities that support the pull strategy of research use by policy-makers. The funding of research centres or the commissioning of particular research projects are included in this factor, together with standard procedures for commissioning research. The division between ‘funding and commissioning of research’ under this subcategory, and the factor ‘government and academia collaborative research’ under the ‘inter-organisational communication and collaboration’ subcategory, is a distinction between the pull strategy of research by policy organisations and an interactive approach to research generation.

Many studies mentioned political will and support of research as facilitating research use, but often without further clarification. When clarified, the legislative support of the implementation of evidence-informed policies and for the development of internal capacities for evidence-informed policy-making were mentioned as supporting factors [16, 77]. Additionally, Tabak et al. [45] found that political support was considered important for local governmental officials when developing sustainable policies and programmes. They also found that political support was existing in local health departments that had high capacity in developing sustainable policies and programmes, and the support was lacking in departments with low capacity.

The factor ‘establishing platforms for engaging all stakeholders across sectors in policy discussions’ was identified in two reviews and two empirical studies, where research evidence is discussed with all stakeholders to find a consensus about the available research evidence. One review and four empirical studies provided support for the importance of an ‘open and transparent policy-making process that creates opportunities for public input and transparent policy procedures’.

The scoping review findings show that external research is mostly used during agenda-setting, when it is openly a subject of discussion by all stakeholders. The outcomes also indicate that research integration would benefit if this process is led by policy organisations. Strong leadership guiding the research integration (or knowledge translation) process is paramount to effective research use in policy organisations. This means that policy organisations should have internal research capacity, research on policy know-how, and coordination capacity to involve external stakeholders in each policy-making phase.

The subcategory of ‘implicit rules and preferences on how to make policy’ encompasses factors that are linked to the implicit rules of conduct and the preferred traits of organisational members; it is embedded in the organisation’s culture. We used the term ‘value’ to cover both rules of conduct and preferred traits. Two factors have been identified as highly supported by research under this subcategory. The factor ‘high value placed on rationality, professionalism, speciality, measurement, evaluation and quality improvement’ is closely associated with a rational action approach and new public management. This factor contains the view on professionalism and the integration of a rational approach in a not-so-rational policy process, which is believed to be the ability to balance “*gold standard systematic reviews with pragmatic, rapid reviews that gain in timeliness and accessibility what they lose in depth and detail*” [76].

The second most highly research-supported factor in this subcategory is the ‘shared importance and high value of research in policy-making as part of the organisation’s culture’. This factor includes a direct focus on the significance and value of research use as a fundamental part of policy-making. This entails shared agreements and commitment among the organisation’s members about the importance and value of research findings; this is supported by peers’ endorsement of research and operationalising commitment. This factor is directly linked to ‘efforts to create an organisational culture favouring research use’, which falls under the ‘strategic commitment toward research use’ subcategory.

#### Organisational characteristics

The general category of ‘organisational characteristics’ covers factors associated with the characteristics of the organisation (such as its size and functioning, policy-making level and geographic location). Only nine studies have contributed to this category, and none of the factors are highly supported by research (Fig. 3). The primary contributing study examined policy-makers at different levels in diverse sectors, without providing a clear definition other than people identified as making policy-related decisions. The rest of the studies investigated civil servants at different policy levels, with a slightly increased focus on multi-level policy-making.

Despite the lack of research in this overall category, several factors seem worth investigating further (e.g. the location of policy organisations as well as the complexity and size of organisations). However, for several factors in this overall category, further debate is needed on the usefulness of conducting research in this area.

## Discussion

This scoping review identified 14 reviews and 40 empirical studies that included organisational factors used in facilitating research use in policy organisations. These studies allowed us to identify some overarching themes concerning organisational factors that positively affect research use. Furthermore, we were able to identify 64 organisational factors that facilitate research use. Out of these 64 factors, 27 were reported by seven or more studies, including at least one review. We developed a conceptual map of the organisational factors that provides a comprehensive overview of the organisational factors. This map clearly demonstrates a close link between individual factors and organisational factors in relation to research use.

Our synthesis confirms the importance of an intra-organisational perspective when exploring research use. This shows that many organisational factors are critical facilitators of research use. It also shows that many factors and mechanisms are understudied [18].

Few studies provided comprehensive theoretical application for hypothesis testing, making better study designs with theory application and testing in this area pertinent. Additionally, the studies seldom included politicians as the study population, which we believe are important members of the policy organisation. Therefore, we would like to encourage a discussion about who are members of policy organisations in order to fully grasp the organisational factors of research use for policy-making.

### Empirical reflections

The review findings indicate that policy organisations are very sensitive towards external factors such as the institutional arrangements in the country, external policy actors (including funding agencies), collaboration with other institutions and public opinion. Therefore, research use within policy organisations should not be examined in a vacuum, but rather, the organisational factors should be framed in such a way that this sensitivity to external factors is depicted.

Policies are informed by both internal and external research. Internal research activities are integrated into policy more easily than external research (e.g. systematic reviews). Future research could benefit from distinguishing between internal and external investigations and the different types of studies used for policy-making.

Factors related to the creation of systems and a good infrastructure for research use have shown much promise. Focus has primarily been on access to repositories of external research whereby other facilitating factors seem necessary to explore further (e.g. tailored dissemination, personal access to an internal expert and comprehensive knowledge management systems). Additionally, the results related to the management of research integration indicate that an internal management of capacity-building, functional research integrating procedures, performances and organisational structures are some of the key ingredients in successful research integration strategies.

The creation of a political environment that aids research use is often an area outside organisational management; however, it seems there may be several intra-organisational factors that can influence perceptions of research among politicians. Therefore, this is not a top-down stream of influence (e.g. cross-sector collaboration and influential members of an organisation). For instance, when reflecting on the ‘political environment’ subcategory from the angle of organisational culture, despite the lack of sufficient evidence, it seems that the political environment is a precursor to the formation of implicit rules and preferences favouring research use. However, evidence is still needed on the role of politicians in developing evidence-informed policies and how to increase preferences for research among politicians.

Many of the empirical studies depicted the complexity of research use when it enters the political arena and competes with other kinds of evidence. However, some studies showed promise through applying open and transparent processes when introducing and integrating research [52, 53, 59, 60]. Our scoping review findings related to low-resource countries demonstrated that international donors and partners (such as the WHO) are more active stakeholders in getting research evidence on the policy agenda than in high-resource countries. This fact highlights the significance of partnerships in places where research evidence can be discussed, research priorities identified and consensus about the evidence built. However, further examination is needed to advance our understanding of the role of the members of political organisations in coordinating structured and trusted partnerships as well as more open and transparent discussions about research in different policy contexts. Based on the importance of individuals’ ‘competences and being able to champion research use in a political setting’, one might further argue that there is a need for more evidence to facilitate understanding of which strategies are most effective in integrating research into policy decision-making that go beyond just improving research capacity.

In relation to the individual factors, our findings support the need to look into age-related factors to find better reasons for research use. One example of such a factor would be the association between a high decision-making authority and research use on the one hand and incentives for younger or recently graduated policy-makers to use research on the other.

For the development of strategies to increase research use, we would like to underscore the seriousness of assessing the policy environment and organisational culture before implementing strategies for research use. Moreover, keeping the focus on actual policy processes and the need for research to support policy-makers in fulfilling their tasks shows much promise.

### Theoretical reflections

Even though many of the factors enabling research use are linked to a traditional rational action approach (e.g. evidence-based policy), other facilitating factors are linked to a more flexible approach toward research integration (e.g. EIDM), where studies from different sources and types are mixed together to fit particular policy needs and the political environment.

This review reveals that, in the field of policy-making and organisational research use, DoI theory has been harnessed to examine the use of external research products (e.g. systematic reviews) and to design knowledge translation interventions. However, with so few studies identified and their different applications of the DoI theory, it is important to make further theoretical contributions to this theory, for instance, to test its applicability for internal research generation and knowledge exchange as well as its ability to capture the political aspects of research use in diffusion processes.

The SPIRIT Action Framework stems from a multi-component intervention trial, which has allowed extensive time and effort to go into developing and testing both the framework and several intervention tools and measures of effectiveness [15]. This framework therefore seems to be the most comprehensive and applicable framework for interventions within the field of evidence-informed policy-making, which includes organisational factors. However, the framework and tools developed within it are still being tested for further validation [82].

Our synthesis of the literature reveals a lack of political theories in studies on research use and evidence-based/informed policy-making. This was also recently stressed by Cairney [83], who suggested several theories to comprehend research use in policy-making. Best and Holmes [84] provided another interesting perspective in understanding research in policy organisations through complex actors and systems thinking.

The framework provided by Contandriopoulos et al. [12] on “*collective-level knowledge use*” provides a good basis for further research into policy organisations. However, Contandriopoulos et al. refrained from elaborating on the cultural components intertwined with the institutional rules, norms and procedures influencing research use in policy areas. Nonetheless, they have synthesised a wide range of empirical knowledge and theories that deepen understanding of the complex relationship(s) between policy and research, including theories useful for grasping the intra-organisational perspective on research use.

We found the functional roles of research by Lomas and Brown to be very useful in terms of practically understanding the different functions and roles research has when supporting various kinds of policy decisions. Furthering our understanding in this area can support researchers in becoming more helpful to policy organisations. Political uses of external research are most apparent during agenda-setting. In this regard, it is critical to explore the effectiveness of lobby strategies by researchers in increasing the influence of their research on policy. It is also important to deepen understanding of politicians’ judgement criteria of research evidence, and to what extent research evidence should abide by the same principles as other kinds of proof when being considered in a public policy-making process.

Despite increased efforts to update the existing evidence and theory basis of research utilisation, we still need frameworks that combine different approaches and theories to help us grasp the complex organisational mechanisms that facilitate research use in policy settings. This can aid us in developing causal models for interventions, aiming to expand research use in policy-making [17, 52].

We see great potential in more research applying theoretical lenses based on political and organisational culture theories. Such research might add useful contextual comprehension of research use and helpful intervention strategies for employing research in policy organisations.

### Study strengths and limitations

Using a scoping review methodology, we were able to extensively map the intra-organisational factors of research use in policy-making (including individual factors) and empirically validate theories in this area.

For our exploratory mapping exercise, we narrowed our search down to three databases. However, we are confident that the large number of included reviews provided a good basis for our mapping and that our exploratory approach resulted in a generic concept map that can be used to guide further investigations from different research disciplines (such as political and implementation science and organisational studies), thereby adding nuances to our understanding of the causal mechanisms of research use in policy organisations.

A challenging but worthwhile choice was to only focus on the factors facilitating research use. This allowed us to centre on the positive factors and change mechanisms, which are vital to developing causal models for interventions. However, we would like to stress the importance of also identifying barriers related to research use in policy-making processes for intervention purposes.

By applying the thematic content analysis to published material, we embarked on challenging terrain; we acknowledge that the factors reported in the material have already been subject to theoretical reflections, which we were only able to incorporate into our exploratory analysis to a limited degree.

In our study, we encountered several multi-level and multi-faceted constructs such as ‘research receptive culture’, ‘learning organisation’ and ‘readiness for change’. These constructs were difficult to decompose in our pursuit of identifying factors instead of constructs, especially if the authors did not provide sufficient details of the construct and the underlying factors.

## Conclusions

By applying a scoping review methodology and a thematic content analysis to extract data on organisational factors that facilitate research use, we were able to present an extensive number of organisational factors divided into five main categories (i.e. individual factors, the management of research integration, organisational systems and infrastructures of research use, institutional structures and rules for policy-making and organisational characteristics), 18 subcategories and a total of 64 specific factors. In addition to the summary of reported factors, we provided an overview of the characteristics of the included studies, showing that the main contribution of research is from healthcare and population health and that all levels (national/federal, state/regional and district/local) of policy-making have been explored. Very few studies have used politicians as the study population or have not distinguished between elected and non-elected policy-makers. We provided a theoretical outline of the research and demonstrated a wide range of differently applied theories; however, there is a need for more theoretically founded research.

The goal of this synthesis was to create an overview of intra-organisational factors that can be used for further research and to guide the development of theories and causal models for research use in policy organisations. Despite increased efforts to update the existing evidence and theory basis of research utilisation, we still need frameworks that combine different approaches and theories to help us grasp the complex organisational mechanisms that enable research use in policy settings. Among other things, this can help us build causal models for interventions, aiming to increase research use in public health policy-making.

## Supplementary information

**Additional file 1.** Search profiles and results of the block search shown in tables for each electronic database (PubMed, Academic Search Premier, Scopus).

**Additional file 2.** Overview of included studies and number of organisational factors identified in each study. The overview is divided into reviews and empirical studies.

**Additional file 3.** Table presenting an overview of the organisational factors identified in the included studies, and the targeted policy level and study population of each study.

## Data Availability

The extracted data used and/or analysed during the current review are available from the corresponding author on reasonable request.

## Abbreviations

DOI: Diffussion of Innovation
EBDM: Evidence-based decision-making
EIDM: Evidence-informed decision-making
MoH: Ministry of Health

## References

[1] Hamalainen RM, Aro AR, van de Goor I, Lau CJ, Jakobsen MW, Chereches RM, Syed AM, the REPOPA Consortium (2015). Exploring the use of research evidence in health-enhancing physical activity policies. Health Res Policy Sys.

[2] Zardo P, Collie A (2015). Type, frequency and purpose of information used to inform public health policy and program decision-making. BMC Public Health.

[3] Bowen S, Erickson T, Martens PJ, Crockett S (2009). More than “using research”: the real challenges in promoting evidence-informed decision-making. Health Policy.

[4] Bowen S, Zwi AB (2005). Pathways to “evidence-informed” policy and practice: a framework for action. PLoS Med.

[5] Brownson RC, Fielding JE, Maylahn CM (2009). Evidence-based public health: a fundamental concept for public health practice. Annu Rev Public Health.

[6] Ciliska D, Thomas H, Buffet C (2012). An Introduction to Evidence-informed Public Health and a Compendium of Critical Appraisal Tools for Public Health Practice (Revised).

[7] Larocca R, Yost J, Dobbins M, Ciliska D, Butt M (2012). The effectiveness of knowledge translation strategies used in public health: a systematic review. BMC Public Health.

[8] Cherney A, Head B, Povey J, Ferguson M, Boreham P (2015). Use of academic social research by public officials: exploring preferences and constraints that impact on research use. Evid Policy.

[9] Landry R, Lamari M, Amara N (2003). The extent and determinants of the utilization of university research in government agencies. Public Adm Rev.

[10] Zardo P, Collie A, Livingstone C (2015). Organisational factors affecting policy and programme decision making in a public health policy environment. Evid Policy..

[11] Orton L, Lloyd-Williams F, Taylor-Robinson D, O'Flaherty M, Capewell S (2011). The use of research evidence in public health decision making processes: systematic review. PLoS One.

[12] Contandriopoulos D, Lemire M, Denis JL, Tremblay E (2010). Knowledge exchange processes in organizations and policy arenas: a narrative systematic review of the literature. Milbank Q.

[13] Mitton C, Adair CE, McKenzie E, Patten SB, Perry BW (2007). Knowledge transfer and exchange: review and synthesis of the literature. Milbank Q.

[14] Greenhalgh T, Robert G, Macfarlane F, Bate P, Kyriakidou O (2004). Diffusion of innovations in service organizations: systematic review and recommendations. Milbank Q.

[15] Redman S, Turner T, Davies H, Williamson A, Haynes A (2015). The SPIRIT Action Framework: a structured approach to selecting and testing strategies to increase the use of research in policy. Soc Sci Med.

[16] Huckel Schneider C, Campbell D, Milat A, Haynes A, Quinn E. What are the key organisational capabilities that facilitate research use in public health policy? Public Health Res Pract. 2014;25. 10.17061/phrp2511406. 10.17061/phrp2511406 25828445

[17] Moore G, Redman S, Haines M, Todd A (2011). What works to increase the use of research in population health policy and programmes: a review. Evid Policy.

[18] Haynes A, Rowbotham SJ, Redman S, Brennan S, Williamson A, Moore G (2018). What can we learn from interventions that aim to increase policy-makers’ capacity to use research? A realist scoping review. Health Res Policy Sys.

[19] Masood S, Kothari A, Regan S. The use of research in public health policy: a systematic review. Evid Policy. 2018. 10.1332/174426418X15193814624487.

[20] Anderson JE (2011). Public Policymaking: An Introduction.

[21] Langley A, Mintzberg H, Pitcher P, Posda E, Saint-Macary J (1995). Opening up decision making: the view from the black stool. Organ Sci.

[22] March JG, Olsen JP, Rhodes RAW, Binder SA, Rockman BA (2009). Elaborating the “New Institutionalism”. The Oxford Handbook of Political Institutions.

[23] Schein EH, Schein P (2017). Organizational Culture and Leadership.

[24] Arksey H, O'Malley L (2005). Scoping studies: towards a methodological framework. Int J Soc Res Methodol.

[25] The Research Unit for Research Utilisation: Publications & Resources. 2017. http://www.ruru.ac.uk/publications/. Accessed 2 Jul 2017.

[26] Health Evidence: Presentations & Publications. 2017. https://www.healthevidence.org/presentations-publications.aspx. Accessed 2 Jul 2017.

[27] Dobbins M, Cockerill R, Barnsley J, Ciliska D (2001). Factors of the innovation, organization, environment, and individual that predict the influence five systematic reviews had on public health decisions. Int J Tech Assess Health Care.

[28] Hemsley-Brown J (2004). Facilitating research utilisation. A cross-sector review of research evidence. Int J Pub Sector Manag.

[29] Oliver K, Innvar S, Lorenc T, Woodman J, Thomas J (2014). A systematic review of barriers to and facilitators of the use of evidence by policymakers. BMC Health Serv Res.

[30] Peirson L, Ciliska D, Dobbins M, Mowat D (2012). Building capacity for evidence informed decision making in public health: a case study of organizational change. BMC Public Health.

[31] Perrier L, Mrklas K, Lavis JN, Straus SE (2011). Interventions encouraging the use of systematic reviews by health policymakers and managers: a systematic review. Implement Sci.

[32] Tricco AC, Cardoso R, Thomas SM, Motiwala S, Sullivan S (2016). Barriers and facilitators to uptake of systematic reviews by policy makers and health care managers: a scoping review. Implement Sci.

[33] Belkhodja O, Amara N, Landry R, Ouimet M (2007). The extent and organizational determinants of research utilization in Canadian health services organizations. Sci Comm.

[34] Jewell CJ, Bero LA (2008). “Developing good taste in evidence”: facilitators of and hindrances to evidence-informed health policymaking in state government. Milbank Q.

[35] Rogers EM (1995). Diffusion of Innovations.

[36] Armstrong R, Waters E, Dobbins M, Anderson L, Moore L (2013). Knowledge translation strategies to improve the use of evidence in public health decision making in local government: intervention design and implementation plan. Implement Sci.

[37] Schreier M (2014). The SAGE Handbook of Qualitative Data Analysis.

[38] Hardy AK, Nevin-Woods C, Proud S, Brownson RC. Promoting evidence-based decision making in a local health department, Pueblo city-County, Colorado. Prev Chronic Dis. 2015;12. 10.5888/pcd12.140507. 10.5888/pcd12.140507 PMC4492218 26111156

[39] Hawkes S, K Aulakh B, Jadeja N (2016). Strengthening capacity to apply health research evidence in policy making: experience from four countries. Health Policy Plan.

[40] Jacobs JA, Dodson EA, Baker EA, Deshpande AD, Brownson RC (2010). Barriers to evidence-based decision making in public health: a national survey of chronic disease practitioners. Public Health Rep.

[41] Jbilou J, Amara N, Landry R (2007). Research-based-decision-making in Canadian health organizations: a behavioural approach. J Med Syst.

[42] Kothari A, Edwards N, Hamel N, Judd M (2009). Is research working for you? Validating a tool to examine the capacity of health organizations to use research. Implement Sci.

[43] Oh CH, Rich RF (1996). Explaining use of information in public policymaking. Knowl Policy.

[44] Percy-Smith J, Speller V, Nutley SM (2006). Evidence Informed Policy and Practice: A Review of Approaches Used in Health Improvement in Scotland.

[45] Tabak RG, Duggan K, Smith C, Aisaka K, Moreland-Russell S (2016). Assessing capacity for sustainability of effective programs and policies in local health departments. J Public Health Manag Pract.

[46] Yost J, Dobbins M, Traynor R, DeCorby K, Workentine S, Greco L (2014). The usefulness of having processes and tools in place for evidence-informed public health decision making are part of organizational factors. BMC Public Health.

[47] Zardo P, Collie A (2014). Predicting research use in a public health policy environment: results of a logistic regression analysis. Implement Sci.

[48] Dobbins M, Ciliska D, Cockerill R, Barnsley J, DiCenso A (2002). A framework for the dissemination and utilization of research for health-care policy and practice. Online J Knowl Synth Nurs.

[49] Liverani M, Hawkins B, Parkhurst JO (2013). Political and institutional influences on the use of evidence in public health policy. A systematic review. PLoS One.

[50] Morgan G (2010). Evidence-based health policy: a preliminary systematic review. Health Edu J.

[51] Walter I, Nutley S, Davies H (2005). What works to promote evidence-based practice? A cross-sector review. Evid Policy..

[52] Williamson A, Makkar SR, McGrath C, Redman S (2015). How can the use of evidence in mental health policy be increased? A systematic review. Psych Serv.

[53] Makkar SR, Turner T, Williamson A, Louviere J, Redman S (2016). The development of ORACLe: a measure of an organisation's capacity to engage in evidence-informed health policy. Health Res Policy Syst.

[54] El-Jardali F, Lavis JN, Ataya N, Jamal D, Ammar W (2012). Use of health systems evidence by policymakers in eastern mediterranean countries: views, practices, and contextual influences. BMC Health Serv Res.

[55] Hutchinson E, Parkhurst J, Phiri S, Gibb DM, Chishinga N (2011). National policy development for cotrimoxazole prophylaxis in Malawi, Uganda and Zambia: the relationship between context, evidence and links. Health Res Policy Syst.

[56] van de Goor I, Hamalainen RM, Syed A, Juel Lau C, Sandu P (2017). Determinants of evidence use in public health policy making: results from a study across six EU countries. Health Policy.

[57] von Lengerke T, Rütten A, Vinck J, Abel T, Kannas L (2004). Research utilization and the impact of health promotion policy. Int J Public Healt..

[58] Laws R, King L, Hardy LL, Milat A, Rissel C (2013). Utilization of a population health survey in policy and practice: a case study. Health Res Policy Sys..

[59] Nabyonga-Orem J, Ssengooba F, Macq J, Criel B (2014). Malaria treatment policy change in Uganda: what role did evidence play?. Malar J.

[60] van der Arend J (2014). Bridging the research/policy gap: policy officials’ perspectives on the barriers and facilitators to effective links between academic and policy worlds. Policy Stud.

[61] Wye L, Brangan E, Cameron A, Gabbay J, Klein JH (2015). Evidence based policy making and the ‘art’ of commissioning - How English healthcare commissioners access and use information and academic research in ‘real life’ decision-making: an empirical qualitative study. BMC Health Serv Res.

[62] Innvaer S, Vist G, Trommald M, Oxman A (2002). Health policy-makers’ perceptions of their use of evidence: a systematic review. J Health Serv Res Policy.

[63] Nabyonga JO, Mafigiri DK, Marchal B, Ssengooba F, Macq J, Criel B (2012). Research, evidence and policymaking: the perspectives of policy actors on improving uptake of evidence in health policy development and implementation in Uganda. BMC Public Health.

[64] Lomas J, Brown AD (2009). Research and advice giving: a functional view of evidence-informed policy advice in a Canadian ministry of health. Milbank Q.

[65] Trostle J, Bronfman M, Langer A (1999). How do researchers influence decision-makers? Case studies of Mexican policies. Health Policy Plan.

[66] Newman J (2014). Revisiting the “two communities” metaphor of research utilisation. Int J Public Sector Manag.

[67] Albert MA, Fretheim A, Maïga D (2007). Factors influencing the utilization of research findings by health policy-makers in a developing country: the selection of Mali's essential medicines. Health Res Policy Syst.

[68] Atkins L, Kelly MP, Littleford C, Leng G, Michie S (2017). Reversing the pipeline? Implementing public health evidence-based guidance in english local government. Implement Sci.

[69] Imani-Nasab MH, Seyedin H, Majdzadeh R, Yazdizadeh B, Salehi M (2014). Development of evidence-based health policy documents in developing countries: a case of Iran. Glob J Health Sci.

[70] Nutley S, Walter I, Bland N (2002). The institutional arrangements for connecting evidence and policy: the case of drug misuse. Public Policy Adm.

[71] Twose C, Swartz P, Bunker E, Roderer NK, Oliver KB (2008). Public health practitioners’ information access and use patterns in the Maryland (USA) public health departments of Anne Arundel and Wicomico Counties. Health Info Libraries J.

[72] Oh CH (1996). Information searching in governmental bureaucracies: an integrated model. Am Rev Public Adm.

[73] Brennan SE, Cumpston M, Misso ML, McDonald S, Murphy MJ (2016). Design and formative evaluation of the Policy Liaison Initiative: a long-term knowledge translation strategy to encourage and support the use of Cochrane systematic reviews for informing health policy. Evid Policy.

[74] Elliott H, Popay J (2000). How are policy makers using evidence? Models of research utilisation and local NHS policy making. J Epidemiol Community Health.

[75] Larsen M, Gulis G, Pedersen KM (2012). Use of evidence in local public health work in Denmark. Int J Public Health.

[76] Reul NK (2015). Introduction to evidence-based decision making in a public workers’ compensation agency. Phys Med Rehabil Clin N Am.

[77] Mwendera CA, De Jager C, Longwe H, Phiri K, Hongoro C (2016). Facilitating factors and barriers to malaria research utilization for policy development in Malawi. Malar J.

[78] Fazli GS, Creatore MI, Matheson FI, Guilcher S, Kaufman-Shriqui V (2017). Identifying mechanisms for facilitating knowledge to action strategies targeting the built environment. BMC Public Health.

[79] Haynes A, Brennan S, Carter S, O'Connor D, Schneider CH (2014). Protocol for the process evaluation of a complex intervention designed to increase the use of research in health policy and program organisations (the SPIRIT study). Implement Sci.

[80] Maybin J (2015). Policy analysis and policy know-how: a case study of civil servants in England's department of health. J Comp Policy Analysis.

[81] Dobbins M, Robeson P, Ciliska D, Hanna S, Cameron R (2009). A description of a knowledge broker role implemented as part of a randomized controlled trial evaluating three knowledge translation strategies. Implement Sci.

[82] Makkar SR, Haynes A, Williamson A, Redman S (2018). Organisational capacity and its relationship to research use in six Australian health policy agencies. PLoS One.

[83] Cairney P (2016). The Politics of Evidence-based Policy Making.

[84] Best A, Holmes B (2010). Systems thinking, knowledge and action: towards better models and methods. Evid Policy..

