# Supplementary material for: Organisational factors that facilitate research use in public health policy-making: a scoping review
Source: Health Res Policy Syst. 2019 Nov 21;17:90. doi: 10.1186/s12961-019-0490-6 (PMC6869261; doi:10.1186/s12961-019-0490-6)
Supplement: Supplementary file 1 — Additional file 1. Search profiles and results of the block search shown in tables for each electronic database (PubMed, Academic Search Premier, Scopus). [file 12961_2019_490_MOESM1_ESM.pdf]

Additional file 1 Search profiles and results

Search profile for PubMed 04.04.2017-05.04.2017

Focus 1: Target population - policymakers	Hits	Focus 2: Intervention - Research evidence use	Hits	Focus 3: Policy area - HEPA policy	Hits	Focus 4: Theory	Hits
organization* [tiab]	315048	"evidence based"[tiab]	82325	Health Services Research [MesH Terms]	142642	Models, Organizational[MesH Terms]	17131
organisation*[tiab]	38571	"evidence informed"[tiab]	1212	Public Policy[MesH Terms]	122596	framework*[tiab]	192807
institution*[tiab]	214699	"research based"[tiab]	3528	Health Policy[MesH Terms]	92483	theor*[tiab]	518157
administrat*[tiab]	798070	"research informed"[tiab]	182	health promotion polic*[tiab]	221	approach*[tiab]	1374170
agen*[tiab]	935354	"evidence use"[tiab]	61	public health polic*[tiab]	4356	model[tiab]	1637253
"public official"[tiab]	27	"use of evidence"[tiab]	1668	"health policy"[tiab]	16918	models[tiab]	714377
"public officials"[tiab]	263	"using evidence"[tiab]	0	"health policies"[tiab]	4633	"best practice"[tiab]	9111
"civil servant"[tiab]	133	"evidence translation"[tiab]	36	"planning policy"[tiab]	891	determin*[tiab]	2970496
"civil servants"[tiab]	996	"translating evidence"[tiab]	285	"planning policies"[tiab]	195	pathway*	857241
policymaker*[tiab]	7416	"translation of evidence"[tiab]	0	"health service"[tiab]	37961	concept*[tiab]	388302
"policy maker"[tiab]	220	"evidence utilization"[tiab]	19	"health services"[tiab]	64084	Benchmarking[MesH Terms]	11429
"policy makers"[tiab]	15828	"utilizing evidence"[tiab]	54	physical activity polic*[tiab]	131	review[tiab]	1218545

administrative personnel[MesH Terms]	38021	"utilization of evidence"[tiab]	0	sedentary lifestyle polic*[tiab]	0	overview[tiab]	124573
department*[tiab]	252615	"evidence utilisation"[tiab]	8	physical exercise* polic*[tiab]	3912	mechanism*	1767445
government[MesH Terms]	133707	"utilising evidence"[tiab]	0	sports polic*[tiab]	7		
government*[tiab]	83079	"utilisation of evidence"[tiab]	0	sport polic*[tiab]	15		
"policy advisor"[tiab]	5	"evidence uptake"[tiab]	36	healthy lifestyle polic*[tiab]	3		
"policy advisors"[tiab]	40	"uptake of evidence"[tiab]	0	healthy public polic*[tiab]	208		
"policy analyst"[tiab]	30	"evidence impact"[tiab]	5	"public policy"[tiab]	7950		
"policy analysts"[tiab]	227	"impact of evidence"[tiab]	0	"public policies"[tiab]	2013		
Organization and Administration[MesH Terms]	1212651	"evidence dissemination"[tiab]	13				
Government Employees[MesH Terms]	12	"disseminating evidence"[tiab]	113				
Public Health Administration[MesH Terms]	14755	"dissemination of evidence"[tiab]	0				
policymaking[tiab]	988	"evidence diffusion"[tiab]	0				
"policy making"[tiab]	3423	"diffusion of evidence"[tiab]	0				
Policy Making [MesH Terms]	21945	"evidence exchange"[tiab]	0				
decision making[MesH Terms]	164682	"exchanging evidence"[tiab]	0				
"decision making"[tiab]	95984	"exchange of evidence"[tiab]	0				
		"evidence transfer"[tiab]	8				
		"transferring evidence"[tiab]	18				
		"transfer of evidence"[tiab]	0				

		"evidence capacity"[tiab]	0				
		"research use"[tiab]	873				
		"use of research"[tiab]	0				
		"using research"[tiab]	0				
		"research translation"[tiab]	166				
		"translating research"[tiab]	645				
		"translation of research"[tiab]	483				
		"research utilization"[tiab]	608				
		"utilizing research"[tiab]	47				
		"utilization of research"[tiab]	127				
		"research utilisation"[tiab]	71				
		"utilising research"[tiab]	0				
		"utilisation of research"[tiab]	0				
		"research uptake"[tiab]	47				
		"uptake of research"[tiab]	0				
		"research impact"[tiab]	202				
		"impact of research"[tiab]	0				
		"research dissemination"[tiab]	148				
		"disseminating research"[tiab]	122				
		"dissemination of research"[tiab]	271				
		"research diffusion"[tiab]	0				
		"diffusion of research"[tiab]	0				
		"research transfer"[tiab]	35				
		"transferring research"[tiab]	35				
		"transfer of research"[tiab]	0				
		"research capacity"[tiab]	1004				
		"research literacy"[tiab]	62				
		"knowledge use"[tiab]	88				
		"use of knowledge"[tiab]	0				

		"using knowledge"[tiab]	0				
		"knowledge translation"[tiab]	1888				
		"translating knowledge"[tiab]	166				
		"translation of knowledge"[tiab]	0				
		"knowledge utilization"[tiab]	66				
		"utilizing knowledge"[tiab]	32				
		"utilization of knowledge"[tiab]	0				
		"knowledge utilisation"[tiab]	9				
		"utilising knowledge"[tiab]	6				
		"utilisation of knowledge"[tiab]	0				
		"knowledge dissemination"[tiab]	169				
		"disseminating knowledge"[tiab]	92				
		"dissemination of knowledge"[tiab]	0				
		"knowledge diffusion"[tiab]	35				
		"diffusing knowledge"[tiab]	0				
		"diffusion of knowledge"[tiab]	64				
		"knowledge exchange"[tiab]	396				
		"exchanging knowledge"[tiab]	24				
		"exchange of knowledge"[tiab]	181				
		"knowledge sharing"[tiab]	506				
		"sharing of knowledge"[tiab]	0				
		"scientific information use"[tiab]	0				
		"using scientific information"[tiab]	0				

		"use of scientific information"[tiab]	0				
		"information literacy"[tiab]	303				
		"science literacy"[tiab]	62				
		"scientific literacy"[tiab]	130				
		"scientific capacity"[tiab]	47				
		"science capacity"[tiab]	11				
Block search	3639208		93327		2300011		8467693
Focus 1 AND 2	39279						
Focus 1 AND 2 AND 3	4323						
Focus 1 AND 2 AND 3 AND 4	3033						

Full search strategy for PubMed:

((((((((((((((((((((((((((((((((((organization*[Title/Abstract]) OR organisation*[Title/Abstract]) OR institution*[Title/Abstract]) OR administrat*[Title/Abstract]) OR agen*[Title/Abstract]) OR "public official"[Title/Abstract]) OR "public officials"[Title/Abstract]) OR "civil servant"[Title/Abstract]) OR "civil servants"[Title/Abstract]) OR policymaker*[Title/Abstract]) OR "policy maker"[Title/Abstract]) OR "policy makers"[Title/Abstract]) OR administrative personnel[MeSH Terms]) OR department*[Title/Abstract]) OR government[MeSH Terms]) OR government*[Title/Abstract]) OR "policy advisor"[Title/Abstract]) OR "policy advisors"[Title/Abstract]) OR "policy analyst"[Title/Abstract]) OR "policy analysts"[Title/Abstract]) OR (organization and administration[MeSH Terms])) OR government employees[MeSH Terms]) OR administration, public health[MeSH Terms]) OR policymaking[Title/Abstract]) OR "policy making"[Title/Abstract]) OR policy making[MeSH Terms]) OR decision making[MeSH Terms]) OR "decision making"[Title/Abstract])) AND (("evidence based"[Title/Abstract]) OR "evidence informed"[Title/Abstract]) OR "research based"[Title/Abstract]) OR "research informed"[Title/Abstract]) OR "evidence use"[Title/Abstract]) OR "use of evidence"[Title/Abstract]) OR "using evidence"[Title/Abstract]) OR "evidence translation"[Title/Abstract]) OR "translating evidence"[Title/Abstract]) OR "translation of evidence"[Title/Abstract]) OR "evidence utilization"[Title/Abstract]) OR "utilizing evidence"[Title/Abstract]) OR "utilization of evidence"[Title/Abstract]) OR "evidence utilisation"[Title/Abstract]) OR "utilising evidence"[Title/Abstract]) OR "utilisation of evidence"[Title/Abstract]) OR "evidence uptake"[Title/Abstract]) OR "uptake of evidence"[Title/Abstract]) OR "evidence impact"[Title/Abstract]) OR "impact of evidence"[Title/Abstract]) OR "evidence dissemination"[Title/Abstract]) OR "disseminating

evidence"[Title/Abstract]) OR "dissemination of evidence"[Title/Abstract]) OR "evidence diffusion"[Title/Abstract]) OR "diffusion of evidence"[Title/Abstract]) OR "evidence exchange"[Title/Abstract]) OR "exchanging evidence"[Title/Abstract]) OR "exchange of evidence"[Title/Abstract]) OR "evidence transfer"[Title/Abstract]) OR "transferring evidence"[Title/Abstract]) OR "transfer of evidence"[Title/Abstract]) OR "evidence capacity"[Title/Abstract]) OR "research use"[Title/Abstract]) OR "use of research"[Title/Abstract]) OR "using research"[Title/Abstract]) OR "research translation"[Title/Abstract]) OR "translating research"[Title/Abstract]) OR "translation of research"[Title/Abstract]) OR "research utilization"[Title/Abstract]) OR "utilizing research"[Title/Abstract]) OR "utilization of research"[Title/Abstract]) OR "research utilisation"[Title/Abstract]) OR "utilising research"[Title/Abstract]) OR "utilisation of research"[Title/Abstract]) OR "research uptake"[Title/Abstract]) OR "uptake of research"[Title/Abstract]) OR "research impact"[Title/Abstract]) OR "impact of research"[Title/Abstract]) OR "research dissemination"[Title/Abstract]) OR "disseminating research"[Title/Abstract]) OR "dissemination of research"[Title/Abstract]) OR "research diffusion"[Title/Abstract]) OR "diffusion of research"[Title/Abstract]) OR "research transfer"[Title/Abstract]) OR "transferring research"[Title/Abstract]) OR "transfer of research"[Title/Abstract]) OR "research capacity"[Title/Abstract]) OR "research literacy"[Title/Abstract]) OR "knowledge use"[Title/Abstract]) OR "use of knowledge"[Title/Abstract]) OR "using knowledge"[Title/Abstract]) OR "knowledge translation"[Title/Abstract]) OR "translating knowledge"[Title/Abstract]) OR "translation of knowledge"[Title/Abstract]) OR "knowledge utilization"[Title/Abstract]) OR "utilizing knowledge"[Title/Abstract]) OR "utilization of knowledge"[Title/Abstract]) OR "knowledge utilisation"[Title/Abstract]) OR "utilising knowledge"[Title/Abstract]) OR "utilisation of knowledge"[Title/Abstract]) OR "knowledge dissemination"[Title/Abstract]) OR "disseminating knowledge"[Title/Abstract]) OR "dissemination of knowledge"[Title/Abstract]) OR "knowledge diffusion"[Title/Abstract]) OR "diffusing knowledge"[Title/Abstract]) OR "diffusion of knowledge"[Title/Abstract]) OR "knowledge exchange"[Title/Abstract]) OR "exchanging knowledge"[Title/Abstract]) OR "exchange of knowledge"[Title/Abstract]) OR "knowledge sharing"[Title/Abstract]) OR "sharing of knowledge"[Title/Abstract]) OR "scientific information use"[Title/Abstract]) OR "using scientific information"[Title/Abstract]) OR "use of scientific information"[Title/Abstract]) OR "information literacy"[Title/Abstract]) OR "science literacy"[Title/Abstract]) OR "scientific literacy"[Title/Abstract]) OR "scientific capacity"[Title/Abstract]) OR "science capacity"[Title/Abstract]))) AND (((((((((((((((public policy[MeSH Terms]) OR health policy[MeSH Terms]) OR health promotion polic*[Title/Abstract]) OR public health polic*[Title/Abstract]) OR "health policy"[Title/Abstract]) OR "health policies"[Title/Abstract]) OR "planning policy"[Title/Abstract]) OR "planning policies"[Title/Abstract]) OR "health service"[Title/Abstract]) OR "health services"[Title/Abstract]) OR physical activity polic*[Title/Abstract]) OR sports polic*[Title/Abstract]) OR sport polic*[Title/Abstract]) OR healthy lifestyle polic*[Title/Abstract]) OR healthy public polic*[Title/Abstract]) OR "public policy"[Title/Abstract]) OR "public policies"[Title/Abstract]))) AND (((((((((((((models, organizational[MeSH Terms]) OR framework*[Title/Abstract]) OR theor*[Title/Abstract]) OR approach*[Title/Abstract]) OR model[Title/Abstract]) OR models[Title/Abstract]) OR "best practice"[Title/Abstract]) OR determin*[Title/Abstract]) OR pathway*[Title/Abstract]) OR concept*[Title/Abstract]) OR benchmarking[MeSH Terms]) OR review[Title/Abstract]) OR overview[Title/Abstract]) OR mechanism*[Title/Abstract]))

Search profile for Academic Search Premier 28.06.2017-04.07.2017

Advanced search filters: Published date 01.01.1970 – 04.07.2017, abstract or author supplied abstract							
Focus 1: Target population - policymakers	Hits	Focus 2: Intervention - Research evidence use	Hits	Focus 3: Policy area - HEPA policy	Hits	Focus 4: Theory	Hits
organi?ation*	529014	"evidence based"	45901	health promotion polic*	593		
institution*	284969	"evidence informed"	829	public health polic*	7057	framework*	361278
administrat*	526173	"research based"	5636	"health policy"	10670	theor*	1119784
department*	340713	"research informed"	313	"health policies"	3223	approach*	1368031
government*	586334	"evidence use"	96	"planning policy"	679	model OR models	2275950
agen*	694703	"use of evidence"	1966	"planning policies"	508	"best practice"	8738
official OR officials	181337	"using evidence"	1135	"public policy"	20076	determin*	1994823
servant OR servants	8086	"evidence translation"	21	"public policies"	4535	pathway*	436254
policymaker*	13750	"translating evidence"	85	physical activity polic*	454	concept*	582480
"policy maker"	470	"translation of evidence"	154	sedentary lifestyle polic*	2	benchmark*	55822
"policy makers"	27864	"evidence utilization"	13	physical exercis* polic*	15	review	3383710
"policy analyst"	520	"utilizing evidence"	40	sport* polic*	726	overview	195586
"policy analysts"	549	"utilization of evidence"	91	healthy lifestyle polic*	58	mechanism*	1100112
"decision maker"	3110	"evidence utilisation"	5	healthy public polic*	157	factors	1723030
"decision makers"	14730	"utilising evidence"	9				
"decision making"	100270	"utilisation of evidence"	9				
"policy making"	10935	"evidence uptake"	18				
policymaking	4437	"uptake of evidence"	163				
		"evidence impact"	20				

		"impact of evidence"	156				
		"evidence dissemination"	13				
		"disseminating evidence"	58				
		"dissemination of evidence"	200				
		"evidence diffusion"	2				
		"diffusion of evidence"	29				
		"evidence exchange"	2				
		"exchanging evidence"	0				
		"exchange of evidence"	41				
		"evidence transfer"	6				
		"transferring evidence"	7				
		"transfer of evidence"	79				
		"evidence capacity"	2				
		"research use"	902				
		"use of research"	2603				
		"using research"	593				
		"research translation"	109				
		"translating research"	323				
		"translation of research"	391				
		"research utilization"	262				
		"utilizing research"	39				
		"utilization of research"	152				
		"research utilisation"	41				
		"utilising research"	8				
		"utilisation of research"	23				
		"research uptake"	40				
		"uptake of research"	97				
		"research impact"	530				
		"impact of research"	975				
		"research dissemination"	149				

		"disseminating research"	120				
		"dissemination of research"	328				
		"research diffusion"	15				
		"diffusion of research"	47				
		"research transfer"	37				
		"transferring research"	23				
		"transfer of research"	221				
		"research capacity"	846				
		"research literacy"	56				
		"knowledge use"	235				
		"use of knowledge"	1913				
		"using knowledge"	517				
		"knowledge translation"	932				
		"translating knowledge"	79				
		"translation of knowledge"	113				
		"knowledge utilization"	117				
		"utilizing knowledge"	38				
		"utilization of knowledge"	67				
		"knowledge utilisation"	23				
		"utilising knowledge"	10				
		"utilisation of knowledge"	13				
		"knowledge dissemination"	220				
		"disseminating knowledge"	113				
		"dissemination of knowledge"	380				
		"knowledge diffusion"	123				
		"diffusing knowledge"	10				
		"diffusion of knowledge"	135				
		"knowledge exchange"	714				
		"exchanging knowledge"	37				

		"exchange of knowledge"	395				
		"knowledge sharing"	1714				
		"sharing of knowledge"	546				
		"scientific information use"	5				
		"using scientific information"	2				
		"use of scientific information"	21				
		"information literacy"	3455				
		"science literacy"	495				
		"scientific literacy"	772				
		"scientific capacity"	60				
		"science capacity"	18				
Block search	2834840		73885		2300011		10817594
Focus 1 AND 2	21379						
Focus 1 AND 2 AND 3	837						
Focus 1 AND 2 AND 3 AND 4	638						

Full search strategy for Academic Search Premier:

Block 1 search query	organi?ation* OR institution* OR administrat* OR department* OR government* OR agen* OR (official OR officials) OR (servant OR serva OR policymaker* OR ("policy maker" OR "policy makers") OR ("policy analyst" OR "policy analysts") OR ("decision maker" OR "decision makers") OR "decision making" OR "policy making" OR policymaking

Block 2 search query	"evidence based" OR "evidence informed" OR "research based" OR "research informed" OR "evidence use" OR "use of evidence" OR "usi[illegible] evidence" OR "evidence translation" OR "translating evidence" OR "translation of evidence" OR "evidence utili?ation" OR "utili?ing eviden[illegible] OR "utili?ation of evidence" OR "evidence uptake" OR "uptake of evidence" OR "evidence impact" OR "impact of evidence" OR "evidence dissemination" OR "disseminating evidence" OR "dissemination of evidence" OR "evidence diffusion" OR "diffusion of evidence" OR "evid[illegible] exchange" OR "exchanging evidence" OR "exchange of evidence" OR "evidence transfer" OR "transferring evidence" OR "transfer of evide[illegible] OR "evidence capacity" OR "research use" OR "use of research" OR "using research" OR "research translation" OR "translating research" [illegible] "translation of research" OR "research utili?ation" OR "utili?ing research" OR "utili?ation of research" OR "research uptake" OR "uptake o[illegible] research" OR "research impact" OR "impact of research" OR "research dissemination" OR "disseminating research" OR "dissemination of research" OR "research diffusion" OR "diffusion of research" OR "research transfer" OR "transferring research" OR "transfer of research" [illegible] "research capacity" OR "research literacy" OR "knowledge use" OR "use of knowledge" OR "using knowledge" OR "knowledge translation[illegible] "translating knowledge" OR "translation of knowledge" OR "knowledge utili?ation" OR "utili?ing knowledge" OR "utili?ation of knowledge[illegible] "knowledge dissemination" OR "disseminating knowledge" OR "dissemination of knowledge" OR "knowledge diffusion" OR "diffusing knowledge" OR "diffusion of knowledge" OR "knowledge exchange" OR "exchanging knowledge" OR "exchange of knowledge" OR "knowl[illegible] sharing" OR "sharing of knowledge" OR "scientific information use" OR "using scientific information" OR "use of scientific information" O[illegible] "information literacy" OR "science literacy" OR "scientific literacy" OR "scientific capacity" OR "science capacity"
Block 3 search query	(health promotion polic*) OR (public health polic*) OR ("health policy" OR "health policies") OR ("planning policy" OR "planning policies") [illegible] ("public policy" OR "public policies") OR (physical activity polic*) OR (sedentary lifestyle polic*) OR (physical exercis* polic*) OR (sport* po[illegible] OR (healthy lifestyle polic*) OR (healthy public polic*)
Block 4 search query	framework* OR theor* OR approach* OR (model OR models) OR "best practice" OR determin* OR pathway* OR concept* OR benchmark[illegible] review OR overview OR mechanism*

Search profile for Scopus 04.07.2017-07.07.2017

Advanced search filters: Published date 01.01.1970 - 07.07.2017, abstract							
Focus 1: Target population - policymakers	Hits	Focus 2: Intervention - Research evidence use	Hits	Focus 3: Policy area - HEPA policy	Hits	Focus 4: Theory	Hits
organi?ation*	2407299	"evidence based"	91899	(health promotion polic*)	6765	framework*	1135454
institution*	583008	"evidence informed"	1355	(public health polic*)	47595	theor*	3391961
administrat*	1010874	"research based"	13442	("health policy" OR "health policies")	21943	approach*	4401305
department*	465837	"research informed"	564	("planning policy" OR "planning policies")	2988	(model OR models)	7545653
government*	462464	"evidence use"	167	("public policy" OR "public policies")	35360	"best practice"	57658
agen*	1600652	"use of evidence"	2166	(physical activity polic*)	6563	determin*	6516201
(official OR officials)	106565	"using evidence"	2436	(sedentary lifestyle polic*)	235	pathway*	1024574
(servant OR servants)	8023	"evidence translation"	37	(physical exercis* polic*)	1009	concept*	1655020
policymaker*	25008	"translating evidence"	183	(sport* polic*)	4150	benchmark*	189965
("policy maker" OR "policy makers")	57945	"translation of evidence"	230	(healthy lifestyle polic*)	946	review	1956281
("policy analyst" OR "policy analysts")	1250	"evidence utili?ation"	30	(healthy public polic*)	3204	overview	350770
("decision maker" OR "decision makers")	62165	"utili?ing evidence"	101			mechanism*	3345915
"decision making"	281440	"utili?ation of evidence"	122			factors	4734845
"policy making"	19079	"evidence uptake"	37				
policymaking	5857	"uptake of evidence"	198				

		"evidence impact"	37				
		"impact of evidence"	111				
		"evidence dissemination"	16				
		"disseminating evidence"	121				
		"dissemination of evidence"	313				
		"evidence diffusion"	5				
		"diffusion of evidence"	39				
		"evidence exchange"	9				
		"exchanging evidence"	2				
		"exchange of evidence"	9				
		"evidence transfer"	13				
		"transferring evidence"	16				
		"transfer of evidence"	39				
		"evidence capacity"	5				
		"research use"	5391				
		"use of research"	1228				
		"using research"	1228				
		"research translation"	195				
		"translating research"	508				
		"translation of research"	596				
		"research utili?ation"	690				
		"utili?ing research"	102				
		"utili?ation of research"	211				
		"research uptake"	64				
		"uptake of research"	132				
		"research impact"	652				
		"impact of research"	714				
		"research dissemination"	260				
		"disseminating research"	211				
		"dissemination of research"	570				

		"research diffusion"	37				
		"diffusion of research"	51				
		"research transfer"	87				
		"transferring research"	72				
		"transfer of research"	215				
		"research capacity"	1560				
		"research literacy"	76				
		"knowledge use"	590				
		"use of knowledge"	2004				
		"using knowledge"	2534				
		"knowledge translation"	1743				
		"translating knowledge"	145				
		"translation of knowledge"	217				
		"knowledge utili?ation"	448				
		"utili?ing knowledge"	260				
		"utili?ation of knowledge"	260				
		"knowledge dissemination"	696				
		"disseminating knowledge"	287				
		"dissemination of knowledge"	951				
		"knowledge diffusion"	622				
		"diffusing knowledge"	43				
		"diffusion of knowledge"	400				
		"knowledge exchange"	2365				
		"exchanging knowledge"	177				
		"exchange of knowledge"	886				
		"knowledge sharing"	9652				
		"sharing of knowledge"	1093				
		"scientific information use"	4				
		"using scientific information"	12				
		"use of scientific information"	58				

		"information literacy"	4233				
		"science literacy"	453				
		"scientific literacy"	1025				
		"scientific capacity"	172				
		"science capacity"	29				
Block search	6174145		150196		102459		24582325
Focus 1 AND 2	52671						
Focus 1 AND 2 AND 3	2930						
Focus 1 AND 2 AND 3 AND 4	2300						

<u>Full search strategy for Scopus:</u> N/A
