# Supplementary material for: Organisational factors that facilitate research use in public health policy-making: a scoping review
Source: Health Res Policy Syst. 2019 Nov 21;17:90. doi: 10.1186/s12961-019-0490-6 (PMC6869261; doi:10.1186/s12961-019-0490-6)
Supplement: Supplementary file 2 — Additional file 2. Overview of included studies and number of organisational factors identified in each study. The overview is divided into reviews and empirical studies. [file 12961_2019_490_MOESM2_ESM.pdf]

Additional file 2 Overview of included studies and number of organisational factors identified in each study

Overview of included reviews

ID number	Review author(s) and title	Policy area	Policy level	Organisational setting	Study objectives	Theoretical framework or approach	Research utilisation measures	No of organisational factors identified
#1	Greenhalgh et al. (2004). Diffusion of Innovations in Service Organisations: Systematic Review and Recommendations. United Kingdom.	Health care	National, regional and local	Health services organisations	To systematically review empirical studies within the health sector, as well as overview articles and "landmark" empirical studies from outside the health sector on the diffusion of service innovations. The aim is to provide insight on how to spread and sustain innovations in health service delivery and organisation.	Diffusion of innovation by Rogers [42]	Diffusion/adoption of innovation	18
#2	Makkar et al (2016). The development of ORACLe: a measure of an organisation's capacity to engage in evidence-informed health policy. Australia. (Mixed-method study including literature review and interviews)	Health	International, validated on a national level	Governmental agencies	To develop a measure of an organisation's capacity to engage in evidence-informed health policy through a literature review, and validated through by interview with senior policy makers in Australian governmental agencies (N=9) and further prioritised through a discrete choice experiment of interviews with identified experts in health research and policy (N=24).	SPIRIT Action Framework by Redman et al. [13]	Organisational capacity to use research	16

#3	Tricco et al. (2016). Barriers and facilitators to uptake of systematic reviews by policy makers and health care managers: a scoping review.<br>Canada.	Health care	Federal, provincial, regional and local	National, regional, local governmental authorities and agencies and service delivery organisations	To review empirical studies on the barriers and facilitators to use of systematic reviews by health care managers and policy makers using a scoping review method. The aim is to develop recommendations for systematic review authors and to inform research efforts to develop and test formats for systematic reviews that may optimise uptake.	No framework, only use of the concept knowledge translation without reference.	Use of systematic reviews	12
#4	Oliver et al. (2014). A systematic review of barriers to and facilitators of the use of evidence by policymakers.<br>United Kingdom.	Multiple sectors (not specific health focus)	National, regional and local	National, regional, local governmental authorities and agencies and service delivery organisations	To systematically review empirical studies and systematic reviews about factors affecting the use of evidence in policy. This review aimed to update and expand the previous review by Innvaer et al. (2002) by 1) identifying factors which act as barriers to and facilitators of the use of evidence in public policy, including factors perceived by different stakeholder groups; and 2) describing the focus, methods, populations, and findings of the new evidence in this area.	Evidence-informed policy making (EIPM)/evidence-based policy (EBP) and the importance of context (reference to Dobrow et al. [77]).	Perceptions of barriers and facilitators of evidence use	18

#5	Dobbins et al. (2002). A Framework for the Dissemination and Utilisation of Research for Health-Care Policy and Practice. Canada.	Health care	Local	Services delivery	To synthesise literature from 1) organisational behaviour, culture, and decision making from the management field; and 2) research dissemination, utilisation and evidence-based practice from the health field. The aim is to develop a framework for research dissemination and utilisation that is applicable for health policy and clinical decision-making.	Diffusion of innovation by Rogers [42]	Diffusion of innovation	16
#6	Huckel Schneider et al. (2014). What are the key organisational capabilities that facilitate research use in public health policy? Australia. (Mixed-method study including literature review and survey)	Public health	State/Federal	Governmental agencies	1) To review existing comprehensive reviews to identify organisational capabilities that were reported as having the potential to facilitate research use in policy decision making. 2) To test the validity of the organisational capabilities among selected policy makers; whether these capabilities are relevant, practical and applicable in real world policy settings.	Evidence-informed policy making (EIPM)/evidence-based policy (EBP) and the importance of context (reference to Dobrow, Goel and Upshur [78]), no reference or theory referring to organisational capabilities.	Uptake of research in decision making	9

#7	Contandriopoulos et al. (2010). Knowledge Exchange Processes in Organisations and Policy Arenas: A Narrative Systematic Review of the Literature. Canada.	Multiple sectors (not specific health focus)	Unable to extract information	Policy arenas	To systematically survey and review documents that made a core contribution, either conceptually or empirically, to the understanding of collective-level interventions aimed at influencing policymaking or organisational behaviour though knowledge exchange, and especially about the contextual conditions affecting their efficacy. The aim is to strengthen understanding of the processes in such interventions.	Develop of own concept 'collective-level knowledge use', which is the process by which the users incorporate research products into action proposals to influence others' thoughts and practices), multiple references used to support the concept.	Policy process theories/policy communities and networks/knowledge and research utilisation	10
#8	Moore et al. (2011). What works to increase the use of research in population health policy and programmes: a review. Australia.	Population health	National, regional and local	Governmental and non-governmental agencies.	To review empirical studies that evaluate intervention strategies designed to increase the use of evidence from research in population health policy. This review builds on and extends previous analyses (Innvaer et al, 2002; Mitton et al, 2007). The aim is to provide a detailed picture of what is known about the effectiveness of strategies to increase the use of research in policy and the implications for future directions in the development and testing of new intervention strategies in this area.	No theoretical framework used, no explicit approach either.	Effectiveness of strategies to increase the use of research in policy	12

#9	Mitton et al. (2007). Knowledge Transfer and Exchange: Review and Synthesis of the Literature. Canada.	Health	Federal, district and local	Federal, provincial and local health organisations	To examine and summarize the current evidence base for knowledge translation and exchange (KTE) in relation to health policy to create an evidence-based resource for planning KTE processes. The focus is on studies of KTE that could have either an impact on or implications for health care policies at an organisational, regional, provincial, and/or federal level.	Knowledge transfer and exchange approach, which they characterise as an interactive interchange of knowledge between research users and researcher producers, reference to Kiefer et al. [79]	Barriers and facilitators of KTE and effectiveness of KTE interventions on research use in general	9
#10	Walter, Nutley, and Davies (2005). What works to promote evidence-based practice? A cross-sector review. United Kingdom.	Health care, social care, education, criminal justice	National, regional, local	Service organisations	To systematically review current evidence about what works to promote the use of research from across four key sectors: Healthcare, social care, education and criminal justice. The review is an update of a previous systematic review (Walter et al, 2003). The focus is on the key mechanisms that explicitly or implicitly underpin different approaches to implementing evidence-based policy and practice. The review includes only articles that evaluated or reviewed evaluations of interventions to enhance the use of research, leaving out a previous focus on articles concerning barriers and enablers to research use.	Evidence-based policy and practice, and the underpinning mechanisms and resources which leads to change (reference to Pawson [80])	Changes in access to research, knowledge and understanding, attitudes and beliefs, behaviour, outcomes for service users	9

					The aim is to draw out key lessons about effective methods for implementing evidence-based policy and practice.			
#11	Williamson et al. (2015). How Can the Use of Evidence in Mental Health Policy Be Increased? A Systematic Review.<br>Australia.	Mental health	National, regional and local	Regional and local authorities and agencies and service delivery organisations	To systematically review intervention studies that included a component aimed at increasing use of evidence in mental health policy. The aim is to explore what is known about the effectiveness of strategies to increase the use of research in mental health policies.	SPIRIT Action Framework by Redman et al. (citation above)	Effectiveness of intervention strategies to increase research use in policy making or service delivery	7
#12	Liverani, Hawkins, and Parkhurst (2013). Political and Institutional Influences on the Use of Evidence in Public Health Policy. A Systematic Review.<br>United Kingdom.	Public health	International, national, regional and local	Unable to extract information	To systematically review empirical studies that examine the complex interface between politics, policy, and the use of evidence. The aim is to identify what is currently known about the ways in which political factors shape the uptake and use of evidence in health policy making.	EIPM/EBP and the political contestation of policy issues. Reference to Lasswell [81], and Barnes and Parkhurst [82].	Uptake of research in decision making	3
#13	Morgan (2010). Evidence-based health policy: A preliminary systematic review.<br>United Kingdom.	Health	National, regional and local	Federal, provincial and local health organisations	To systematically review published reviews on evidence based health policy. The aim is to identify some of the underpinning factors that promote the development of evidence-based health policy.	Evidence-based health policy (reference to Kemm [83])	Promotion of evidence-based policy	1

#14	Orton et al. (2011). The Use of Research Evidence in Public Health Decision Making Processes: Systematic Review. United Kingdom.	Public health	International, national, regional and local	Public, private, and third sector organisations in a range of sectors pertinent to public health	To synthesise the evidence from empirical studies on how research evidence is used by public health decision makers. The aim is to provide an overview of the extent, type, process of use, other factors influencing the decision-making process; and barriers to and facilitators of the use of research evidence.	EIPM/EBP and the complex environment of decision making.	Extent, type, process of research evidence use, and barriers and facilitators of research evidence use	2

Overview of included empirical studies

ID number	Empirical study authors and title	Study design	Study objective	Organisational setting	Policy area	Policy level	Study population	Theoretical framework or approach	Utilization measures	No of organisational factors identified
15	Albert, Fretheim and Maïga (2007). Factors influencing the utilization of research findings by health policy-makers in a developing country: the selection of Mali's essential medicines. Mali.	Case study	To explore factors influencing research use in developing countries by examining the policy-making process for a pharmaceutical policy common in developing countries; an essential medicines list.	National commission	Health care	National/federal	Civil servants, external policy advisors	Knowledge and research utilisation	Perception of use in the policymaking	6

16	Armstrong et al. (2013). Knowledge translation strategies to improve the use of evidence in public health decision making in local government: intervention design and implementation plan. Australia.	Case study	To develop a knowledge translation intervention for public health decision making in local government.	Local government	Population health	District/local	Civil servants	Diffusion of innovations theory, research utilization theory, and KT frameworks	The impact of the KT intervention on individuals' confidence, skills, and access to RE	6
17	Atkins et al. (2017). Reversing the pipeline? Implementing public health evidence-based guidance in English local government. UK.	Case study	To investigate three aspects of implementing national evidence-based recommendations for public health within a local government context: 1) influences on implementation, 2) how useful guidelines are perceived to be and 3) whether the linear evidence-guidelines-practice model is considered relevant.	Local government	Population health	District/local	Civil servants	Implementati on	Perception of use in the policymaking	3
18	Belkhodja et al. (2007). The extent and organizational determinants of research utilization in Canadian health services organizations. Canada.	Survey	Individual and organisational factors influencing the use of research by managers and professionals in Canadian health service organizations (ministries, regional authorities, and hospitals).	Provincial ministries, governmental agencies, regional health authorities and hospitals	Preventive health and health care	State/regiona l and local	Civil servants	Knowledge and research utilisation	Stages of knowledge utilisation within the last 5 years	9

19	Brennan et al. (2016). Design and formative evaluation of the Policy Liaison Initiative: A long-term knowledge translation strategy to encourage and support the use of Cochrane systematic reviews for informing health policy. Australia.	Case study	To investigate perceptions of the Policy Liaison Initiative (PLI) - a long-term knowledge translation initiative designed to support the use of Cochrane systematic reviews in health policy - and its potential to enable research use.	Governmental agencies	Preventive health and health care	National/federal	Civil servants	The theoretical domains framework (factors from behavioural theory thought to influence professional practice)	Perception of use in the policymaking	12
20	Cherney et al. (2015). Use of academic social research by public officials: Exploring preferences and constraints that impact on research use. Australia.	Survey	To examine how certain preferences, constraints and organisational factors influence the ways in which policy personnel seek out and use academic social research.	State and federal central and line agencies	Multiple sectors (not specific health focus)	State/provincial/regional	Civil servants	Knowledge transfer and exchange	Perceived use of academic products or outputs within the last 12 months to understand policy or programme	7
21	Dobbins et al. (2001). Factors of the innovation, organization, environment, and individual that predict the influence five systematic reviews had on public health decisions. Canada.	Survey	To determine the extent to which five systematic reviews of public health interventions influenced public health decisions and which factors were associated with influencing these decisions.	Regional health unit	Population health	State/regional and local	Civil servants	Diffusion of innovations theory/Research utilisation	Perception of use in the policymaking	7

22	El-Jardali et al. (2012). Use of health systems evidence by policymakers in eastern Mediterranean countries: views, practices, and contextual influences. Algeria, Bahrain, Jordan, Lebanon, Oman, Pakistan, Palestine, Sudan, Tunisia, Yemen.	Survey	To explore policymakers' views and practices regarding the use of health systems evidence in health policymaking in 10 eastern Mediterranean countries, including factors that influence health policymaking and barriers and facilitators to the use of evidence.	Ministries, NGOs, professional associations, and donor agencies	Population health	National/federal	Civil servants, managers from NGOs, professional advocacy groups and donor agencies	Knowledge translation	Perception of use in the policymaking	3
23	Elliott and Popay (2000). How are policy makers using evidence? Models of research utilisation and local NHS policy making. UK.	Case study	To identify factors that facilitate or impede evidence-based policy making at a local level in the UK National Health Service (NHS).	Authority	Health care	State/regional and local	Civil servants	Weiss' problem solving model and Giden's dialogical model of research utilization	Development of guidelines, contracts and user information	5
24	Fazli et al. (2017). Identifying mechanisms for facilitating knowledge to action strategies targeting the built environment. Canada.	Case study	1) To identify the knowledge gaps and other barriers to evidence-based decision-making and policy development related to the built environment 2) To identify the policy development infrastructure, processes and mechanisms needed to drive policy changes in this area.	Ministries, regional and municipal departments, non-profit professional organizations and peer review granting agencies	Built environment (public health, urban planning, and transportation)	National, state/regional and local	Civil servants, external policy advisors	Knowledge to Action Framework	Perception of use in the policymaking	1

25	Hardy et al. (2015). Promoting evidence-based decision making in a local health department, Pueblo city-County, Colorado. USA.	Field/quasi-experiment	To monitor and evaluate the impact of a systematic approach to implement evidence-based decision making in the Pueblo City-County Health Department, Pueblo, Colorado.	Local government	Population health	District/local	Civil servants	Evidence-based decision making/administrative evidence-based practices	Impact of EBDM training on a set of AEBPs and EBDM skills	3
26	Hawkes et al. (2016). Strengthening capacity to apply health research evidence in policy making: experience from four countries. Bangladesh, Gambia, India, Nigeria.	Field/quasi-experiment	1) To strengthening the individual, organizational and institutional capacity of policy makers to use research in low- and middle- income countries. 2) To evaluate five capacity building projects (in Bangladesh, Gambia, India and Nigeria).	Parliament, ministry, health care district/local level health care departments	Health care	National and local	Politicians, civil servants, external policy advisors incl. researchers, others	Evidence-informed policy making/Individual, organizational and institutional capacity (United National Development Programme)	Impact of research capacity building on understanding of process and impact, changes in knowledge, attitudes and practice, and evidence referred to in Parliamentary discussions	3
27	Hutchinson et al. (2011). National policy development for cotrimoxazole prophylaxis in Malawi, Uganda and Zambia: the relationship between context, evidence and links. Malawi, Uganda and Zambia (only data extracted from Malawi).	Case study	To examine the influence of context (including the influence of donor agencies), evidence (both local and international), and the links between researcher, policy makers and those seeking to influence the policy process in three cases of national policy development on cotrimoxazole preventive therapy (CPT).	Ministry	Population health	National/federal	Civil servants, researchers and funding agencies	ODI RAPID Framework for analysing policy making in developing countries in relation to evidence, context, and links	Uptake of research in decision making	2

28	Imani-Nasab et al. (2014). Development of evidence-based health policy documents in developing countries: a case of Iran. Iran.	Case study	To examine the barriers and facilitators in developing evidence-based health policy documents from the perspective of their producers in a developing country.	Ministry	Population health	National/federal	Civil servants	Theory of Planned Behaviour	Production of internal evidence documents	5
30	Jbilou, Amara and Landry (2007). Research-based-decision-making in Canadian health organizations: a behavioural approach. Canada.	Survey	To explore the determinants of research-based-decision-making as a personal behaviour among managers and professionals in health administrations in Canada.	Ministry, Federal and Provincial agencies, regional health service agencies, health care units, and other	Preventive health and health care	National and local	Civil servants	Behavioural theories	Research Based Decision Making (5 levels of adoption)	8
31	Kothari et al. (2009). Is research working for you? Validating a tool to examine the capacity of health organizations to use research. Canada.	Mixed-method study (survey/self-assessment questionnaire and focus group interviews)	1) To determine whether the tool 'Is research working for you? A self-assessment tool and discussion guide for health services management and policy organizations', developed by the Canadian Health Services Research Foundation, demonstrated response variability. 2) To determine how the tool differentiated between organizations that were known to be lower-end or higher-end research users.	Federal government, long-term care organizations, non-governmental organizations, and community-based organizations	Preventive health and health care	National and local	Civil servants and NGO employees	Diffusion of Innovations theory*	Organizational research capacity	4

			3) To determine the potential usability of the tool.							
32	Landry, Lamari and Amara (2003).<br>The Extent and Determinants of the Utilization of University Research in Government Agencies.<br>Canada.	Survey	To analyse the what extent is university research used in government agencies? Are there differences between the policy domains in regard to the extent of use? What determines the use of university research in government agencies?	Governmental agencies	Multiple sectors (not specific health focus)	National and state/regional	Civil servants	Knowledge utilisation	Stages of knowledge utilisation within the last 12 months	6
33	Larsen, Gulis and Pedersen (2012).<br>Use of evidence in local public health work in Denmark.<br>Denmark.	Survey	To investigate how and on which level evidence is used in policy processes related to local public health work in Denmark.	Local government	Population health	District/local	Civil servants	Not Applicable	Perception of use in the policymaking	3
34	Laws et al. (2013).<br>Utilization of a population health survey in policy and practice: A case study.<br>Australia.	Case study	To analyse how the findings from an Australian population monitoring survey series of children's weight and weight-related behaviors (Schools Physical Activity and Nutrition Survey (SPANS)) have been used, and the key facilitators and	National, regional, local governmental authorities and agencies and service delivery organisations	Population health	State/provincial/regional	Civil servants and researchers	Framework by Banzi et al. on five broad research impact categories	Perceptions and documentation of impact on a range of areas incl. policy and practice	7

			barriers to their utilization.							
35	Lomas and Brown (2009).<br>Research and advice giving: a functional view of evidence-informed policy advice in a Canadian Ministry of Health.<br>Australia.	Case study (review of theoretical models and interview with civil servants)	To determine the applicability of evidence-based medicine to health policy based on a review of existing models and exploring the functional roles for research-based evidence in policy advice in the in the Ontario Ministry of Health.	Ministry	Population health	State/provincial/regional	Civil servants	Functional view of Research Use in Policy Framework by Lomas and Brown	Evidence-based policy development	9
36	Mwendera et al. (2016).<br>Facilitating factors and barriers to malaria research utilization for policy development in Malawi.<br>Malawi.	Case study	To assess enhancing factors and barriers of research utilization for malaria policy development in Malawi.	Ministry, National Malarial Control Programme Center, research institutions and stakeholder organisations (only coded for MoH and NMCP)	Population health	National/federal	Civil servants, researchers and international stakeholders (e.g. WHO, USAID, etc.)	The Ottawa Model of Research Use (OMRU) by Logan and Graham guiding the development of KT strategies for the improvement of health service in developing countries	Perceived barriers and facilitators of malaria research utilisation	5

37	Nabyonga-Orem et al. (2014). Malaria treatment policy change in Uganda: what role did evidence play? Uganda.	Case study	To explore the role of evidence, barriers, and factors facilitating the uptake of evidence in the change in malaria treatment policy in Uganda.	Ministry of Health, Medical and drug agencies, district health services clinics	Preventive health and health care	National and local	Civil servants, service managers, clinicians, fields workers, researchers, and other stakeholders	Middle Range Theory outlining the main facilitating factors for translating evidence into health policy in developing countries	Uptake of research in decision making	9
38	Newman (2014). Revisiting the "two communities" metaphor of research utilisation. Australia	Survey	To investigate the two communities' metaphor by comparing the personal and professional characteristics of Australian public servants who claim to use research in their policy work with the characteristics of those who claim not to use research.	Governmental agencies	Multiple sectors (not specific health focus)	State/provincial/regional	Civil servants	The hypothesis of two kind of policy workers by Cunningham and Wescler (2002)	How civil servants value and use academic research in the course of their policy-related work	5
39	Nutley, Walter and Bland (2002). The Institutional arrangements for connecting evidence and policy: the case of drug misuse. UK.	Case study	To examine the development of new institutional arrangements for linking research evidence and policy on drug misuse in England and in Scotland.	Ministry and governmental agencies	Population health	National/federal	Civil servants, service managers, clinicians, fields workers, researchers, and other stakeholders	Six institutional propositions that encourage research use for policy making (condensed from Weiss's ten hypotheses from 1999)	Uptake of research in all stages of policy making from agenda setting/modification of existing policy to implementation of research when implementing the policy	6

40	Oh (1996). Information searching in governmental bureaucracies: An integrated model. USA.	Mixed-method study	To empirically investigate the causality among factors involved in the bureaucratic information searching process and to test an integrated model of information searching that contains four sets of primary variables: decision makers' environments (i.e., nature of policy issues), organization, individual characteristics, and characteristics of information. Based on the conceptual framework, a path model is built and tested against data about knowledge utilization and policy change in two areas of mental health policy (i.e., service and financing).	The Congress, the Department of Health and Human Services, Community Mental Health Centres and other service agencies, and advocacy groups	Mental health	National, state/regional and local	Civil servants, external policy advisors	Integrated model of knowledge utilisation (The rational action approach, the organisational interests approach, the two-communities approach)	Decision makers' search for sources of research information separated into internal and external sources	3
41	Oh and Rich (1996). Explaining use of information in public policymaking. USA.	Survey	Same as above, just with another utilisation measure)	Ministry, federal and state departments, advocacy organizations	Mental health	National, state/regional and local	Civil servants, external policy advisors	Knowledge utilization/Institutional, organizational approach	Reference to different types of research within the past year to help make decisions	3

42	Peirson et al. (2012). Building capacity for evidence informed decision making in public health: a case study of organisational change. Canada.	Case study	To explore and describe critical factors and dynamics in the early implementation of one public health unit's strategic initiative to develop capacity to make evidence-informed decision making standard practice.	Regional health unit	Population health	District/local	Civil servants	Evidence-informed decision making by NCCMT, Integrated knowledge translation, Organisational capacity	State of EIDM activity, activities and dynamics involved in organisational change to promote EIDM, and changes in the presence of evidence and EIDM over time	12
43	Percy-Smith, Speller and Nutley (2006). Evidence informed policy and practice: A review of approaches used in health improvement in Scotland. UK.	Case study	To review the Evidence Informed Policy and Practice (EIPP) initiatives in health improvement in Scotland.	Authority	Preventive health and health care	National and local	Civil servants, external policy advisors	Evidence-informed policy/Knowledge translation/Individual and organisational learning	Perception of use in the policymaking	3
44	Reul (2015). Introduction to evidence-based decision making in a public workers' compensation agency. United States.	Case study	To provide details of several organisational characteristics and process solutions that have permitted the agency to implement evidence-based coverage policies.	State health agency	Population health	State/provincial/regional	Civil servants	Evidence-based/informed policymaking	Evidence-based policy development	7

45	Tabak et al. (2016). Assessing capacity for sustainability of effective programs and policies in local health departments. USA.	Case study	To explore the applicability of the Program Sustainability Framework in high- and low-capacity LHDs as defined by the US national performance standards.	PH Departments	Population health	District/local	Civil servants	Program Sustainability Framework (Organizational capacity, program adaption and evaluation, communications, strategic planning, funding stability, environmental support, partnerships),	Capacity for sustainable EB programs and policies	11
46	Trostle, Bronfman and Langer (1999). How do researchers influence decision-makers? Case studies of Mexican policies. Mexico.	Case study	To reconstruct the processes through which research was used to make decisions and policies; to characterize these processes; and to identify the elements that enable or impede the transfer of research results.	Parliament, ministry, health care district/local level health care departments	Population health	National, state/regional and local	Civil servants, researchers and funding agencies	Framework by Walt and Gilson on how to analyse policy cases according to content, and actors, the process and the context [84].	Factors enabling or promoting and impeding research/policy interactions	3
47	Twose et al. (2008). Public health practitioners' information access and use patterns in the Maryland (USA) public health departments of Anne Arundel and Wicomico Counties.	Mixed-method study	To assess enhancing factors and barriers of research utilisation for malaria policy development in Malawi.	PH Departments	Population health	District/local	Civil servants	Evidence-based public health	Self-reported impacts of literature retrieved through library services on policy decisions	3

	USA.									
48	van de Goor et al. (2017). Determinants of evidence use in public health policy making: Results from a study across six EU countries. Finland, Italy, Romania, UK, The Netherlands, Denmark.	Case study	To map facilitators and barriers in the use of evidence in developing health enhancing physical activity policies in six European countries.	National, regional and local governments, public agencies and other stakeholder agencies	Population health	National, state/regional and local	Civil servants, researchers and other policy actors	Evidence-informed decision making/ policy making	Perceptions of barriers and facilitators of evidence use	8
49	Van der Arend (2014). Bridging the research/ policy gap: policy officials' perspectives on the barriers and facilitators to effective links between academic and policy worlds. Australia.	Mixed-method study (survey and interviews)	To explore the nature, functions and relevance of linkages between academics and public servants in supporting research transfer and uptake. Based on survey and interview data of policy officials' experiences around the availability and use of academic social research.	Governmental agencies	Multiple sectors (not specific health focus)	National and state/regional	Civil servants	Policy and research linkages (multiple references used to support concept). Two-communities metaphor by Caplan (1979) [85] and Wingens (1990) [86]).	Instrumental, conceptual and symbolic use of research, linking relations, and facilitators and barriers to effective linkages	13

50	von Lengerke et al. (2004).<br>Research utilisation and the impact of health promotion policy.<br>Belgium, Finland, Germany, The Netherlands, Spain, Switzerland.	Survey	To test the assumption that research utilisation is positively associated with policy impact only if both political will (i.e., policy opportunities) and social strategies (in intervention and implementation) are present.	Health ministries, local public health departments, public and private health insurance companies, and other service delivery and professional networks	Population health	National, state/regional and local	Policy makers from public, private and third sectors having the professional responsibility for a policy in one's organisation or agency	Two theoretical models of health promotion policy determinants (Richmond & Kotelchuck 1983; Rütten et al. 2000; 2003)	Policy makers report if etiological, behavioural and epidemiologic al research has been takes into account for strategy actions	4
51	Wye et al. (2015).<br>Evidence based policy making and the 'art' of commissioning – how English healthcare commissioners access and use information and academic research in 'real life' decision-making: an empirical qualitative study.<br>UK.	Case study	To learn how academic research can influence policy by exploring health care commissioning in England, commissioners' information seeking behaviour and the role of research in their decisions.	Local NHS Clinical Commissionin g Groups	Health care	District/local	Civil servants and general practitioners (clinical commissioner s)	Evidence-informed decision making/ policy making	Reported and verified use of research evidence in decision making	6
52	Yost et al. (2014).<br>Tools to support evidence-informed public health decision making.<br>Canada.	Case study	To test a seven-step method and tools to develop capacity for evidence-informed decision making.	PH Departments	Population health	District/local	Civil servants	Knowledge transfer and exchange	Perception of use of supporting tools for EIDM	4
53	Zardo and Collie (2014).<br>Predicting research use in a public health policy environment: results of a logistic regression analysis.<br>Australia.	Survey	To quantitatively assess and identify factors that predict research use in specific public health policy environments on the individual, organisational and external level.	Governmenta l agencies	Population health	National/fede ral	Civil servants	Evidence-informed public health policy/Individ ual and organisational factors	Perception of use in the policymaking	3

54	Zardo and Collie (2015).<br>Type, frequency and purpose of information used to inform public health policy and program decision-making.<br>Australia.	Survey	To quantitatively measure the frequency and purpose of use of research evidence in comparison to use of other information types in two government public health agencies (environment, workplace and transport injury prevention and rehabilitation compensation).	Governmental agencies	Population health	National/federal	Civil servants	Evidence-informed decision making/Research translation	Perception of use in the policymaking	3
55	Zardo, Collie and Livingstone (2015).<br>Organisational factors affecting policy and programme decision making in a public health policy environment.<br>Australia.	Field/quasi-experiment	To qualitatively examine the everyday practice of government policy and programme decision making in types in two government public health agencies (same as above).	State health agency	Population health	State/provincial/regional	Civil servants	Evidence-informed decision making/Research translation	Decision making (RE use indirectly)	9
