# Supplementary material for: Organisational factors that facilitate research use in public health policy-making: a scoping review
Source: Health Res Policy Syst. 2019 Nov 21;17:90. doi: 10.1186/s12961-019-0490-6 (PMC6869261; doi:10.1186/s12961-019-0490-6)
Supplement: Supplementary file 3 — Additional file 3. Table presenting an overview of the organisational factors identified in the included studies, and the targeted policy level and study population of each study. [file 12961_2019_490_MOESM3_ESM.pdf]

Additional file 3 Overview of organisational factor and targeted policy level and population in each included study

Study reference (alphabetical order)	Organisational factors as listed in Table 2 in the article	External knowledge exchange	Gender and age	Individual values, interests	Position, status, and role in	Research awareness, research	Performance management	Strategic commitment to	Access to research	Inter-organisational	Intra-organisational	Knowledge management	Political environment	Implicit rules and preferences	Function of the organisation	Size and complexity of the	Policy area	Level of government or	Location	International	National/federal	State/regional	Local	Civil servants	Politicians	Service managers and	Researchers	Other external actors
Albert, Fretheim and Maïga (2007)	8,9,20,22,25,29			√		√	√	√													√			√				√
Armstrong et al. (2013)	22,25,29,30,31,32					√	√	√	√														√	√				
Atkins et al. (2017)	9,15,21			√	√	√																	√	√				
Belkhodja et al. (2007)	2,9,20,25,26,28,29,51,55			√		√	√	√							√	√						√	√	√				
Brennan et al. (2016)	15,16,17,21,25,28,32,33,38, 39,45,50				√	√	√	√	√		√		√	√							√			√				
Cherney et al. (2015)	8,9,18,22,29,31,39			√		√	√	√	√		√											√		√				
Contandriopoulos et al. (2010)	8,9,13,15,28,29,31,34,36,47			√	√			√	√	√			√	√						√	√	√	√	√	√		√	√
Dobbins et al. (2001)	6,9,17,22,25,31,50		√	√		√	√		√					√								√	√	√				

Individual factors
Management of research integration
Organisational systems and infrastructure for research use
Institutional structures and rules for policymaking
Organisational characteristics
Policy level
Population

Study reference (alphabetical order)	Organisational factors as listed in Table 2 in the article	External knowledge exchange	Gender and age	Individual values, interests	Position, status, and role in	Research awareness, research	Performance management	Strategic commitment to	Access to research	Inter-organisational	Intra-organisational	Knowledge management	Political environment	Implicit rules and preferences	Function of the organisation	Size and complexity of the	Policy area	Level of government or	Location	International	National/federal	State/regional	Local	Civil servants	Politicians	Service managers and	Researchers	Other external actors
Dobbins et al. (2002)	3,6,9,14,15,18,23,32,36,39, 46,50,54,55,56,64	√	√	√	√	√	√		√	√	√		√	√	√	√			√				√	√				
El-Jardali et al. (2012)	9,21,25			√		√	√														√			√				√
Elliott and Popay (2000)	16,22,37,39,48					√	√			√	√			√								√	√	√				
Fazli et al. (2017)	36								√	√											√	√	√	√				√
Greenhalgh et al. (2004)	9,12,13,17,21,25,27,28,29,3 0,32,38,39,40,42,45,48,49			√	√	√	√	√	√		√	√	√	√							√	√	√	√		√		
Hardy et al. (2015)	21,25,30					√	√	√															√	√				
Hawkes et al. (2016)	21,35,36					√			√	√											√		√	√	√		√	√
Huckel Schneider et al. (2014)	2,16, 20,21,25,26,43,45,47					√	√					√	√									√		√				
Hutchinson et al. (2011)	12,13				√																√			√			√	√

Individual factors
Management of research integration
Organisational systems and infrastructure for research use
Institutional structures and rules for policymaking
Organisational characteristics
Policy level
Population

Study reference (alphabetical order)	Organisational factors as listed in Table 2 in the article	External knowledge exchange	Gender and age	Individual values, interests	Position, status, and role in	Research awareness, research	Performance management	Strategic commitment to research use	Access to research	Inter-organisational	Intra-organisational	Knowledge management	Political environment	Implicit rules and preferences	Function of the organisation	Size and complexity of the	Policy area	Level of government or	Location	International	National/federal	State/regional	Local	Civil servants	Politicians	Service managers and	Researchers	Other external actors
Imani-Nasab et al. (2014)	9,11,24,25,45			√			√						√								√			√				
Jbilou, Amara and Landry (2007)	3,4,9,15,18,20,29,55	√	√	√	√	√		√								√					√		√	√				
Kothari et al. (2009)	21,25,29,30					√	√	√													√		√	√				√
Landry, Lamari and Amara (2003)	9,18,50,55,61,63			√		√								√		√	√	√			√	√		√				
Larsen, Gulis and Pedersen (2012)	19,20,30					√																	√	√				
Laws et al. (2013)	8,12,30,36,37,45,50			√	√			√		√			√	√								√		√			√	
Liverani, Hawkins and Parkhurst (2013)	8,13,16			√	√	√														√	√	√	√	√	√			√
Lomas and Brown (2009)	22,24,25,27,30,31,34,45,50						√	√	√				√	√								√		√				

Individual factors
Management of research integration
Organisational systems and infrastructure for research use
Institutional structures and rules for policymaking
Organisational characteristics
Policy level
Population

Study reference (alphabetical order)	Organisational factors as listed in Table 2 in the article	External knowledge exchange	Gender and age	Individual values, interests	Position, status, and role in	Research awareness, research	Performance management	Strategic commitment to	Access to research	Inter-organisational	Intra-organisational	Knowledge management	Political environment	Implicit rules and preferences	Function of the organisation	Size and complexity of the	Policy area	Level of government or	Location	International	National/federal	State/regional	Local	Civil servants	Politicians	Service managers and	Researchers	Other external actors
Makkar et al. (2016)	2,21,22,24,25,26,31,32,33,34,36,37,41,44,45,47	√				√	√		√	√		√	√								√			√				√
Mitton et al. (2007)	9,12,13,15,25,27,28,29,50			√	√		√	√						√							√		√	√			√	
Moore et al. (2011)	9,16,17,20,21,22,25,28,29,31,34,50			√		√	√	√	√					√							√	√	√	√		√		√
Morgan (2010)	40										√										√	√	√	√		√	√	√
Mwendera et al. (2016)	22,27,34,36,37						√	√	√	√											√			√			√	√
Nabyonga-Orem et al. (2014)	25,29,36,39,42,44,45,46,60						√	√		√	√	√	√				√				√		√	√		√	√	√
Newman (2014)	4,15,18,20,61		√		√	√											√					√		√				
Nutley, Walter and Bland (2002)	12,16,34,39,40,49				√	√			√		√			√							√			√		√	√	√
Oh (1996)	15,18,26				√	√	√														√	√	√	√				√
Oh and Rich (1996)	9,15,34			√	√				√												√	√	√	√				√

Individual factors
Management of research integration
Organisational systems and infrastructure for research use
Institutional structures and rules for policymaking
Organisational characteristics
Policy level
Population

Study reference (alphabetical order)	Organisational factors as listed in Table 2 in the article	External knowledge exchange	Gender and age	Individual values, interests	Position, status, and role in	Research awareness, research	Performance management	Strategic commitment to	Access to research	Inter-organisational	Intra-organisational	Knowledge management	Political environment	Implicit rules and preferences	Function of the organisation	Size and complexity of the	Policy area	Level of government or	Location	International	National/federal	State/regional	Local	Civil servants	Politicians	Service managers and	Researchers	Other external actors
Oliver et al. (2014)	2,5,7,8,9,11,20,22,23,29,30,31,32,33,34,36,45,47	√	√	√		√	√	√	√	√			√								√	√	√	√		√	√	√
Orton et al. (2011)	25,28						√	√												√	√	√	√	√			√	
Peirson et al. (2012)	9,22,23,25,26,27,28,29,39,41,45,48			√			√	√			√	√	√	√									√	√				
Percy-Smith, Speller and Nutley (2006)	27,36,39							√		√	√										√		√	√				√
Reul (2015)	20,36,42,45,46,49,50											√	√	√								√		√				
Tabak et al. (2016)	16,20,21,23,25,26,27,29,30,38,49					√	√	√						√									√	√				
Tricco et al. (2016)	2,5,8,9,15,17,21,25,29,31,33,50	√	√	√	√	√	√	√	√					√							√	√	√	√		√		
Trostle, Bronfman and Langer (1999)	2,24,46	√					√						√								√	√	√	√			√	√
Twose et al. (2008)	12,25,33				√		√		√														√	√				
van de Goor et al. (2017)	1,2,9,21,25,29,36,37	√		√		√	√	√		√											√	√	√	√			√	√

Individual factors

Management of research integration

Organisational systems and infrastructure for research use

Institutional structures and rules for policymaking

Organisational characteristics

Policy level

Population

Study reference (alphabetical order)	Organisational factors as listed in Table 2 in the article	External knowledge exchange	Gender and age	Individual values, interests	Position, status, and role in	Research awareness, research	Performance management	Strategic commitment to	Access to research	Inter-organisational	Intra-organisational	Knowledge management	Political environment	Implicit rules and preferences	Function of the organisation	Size and complexity of the	Policy area	Level of government or	Location	International	National/federal	State/regional	Local	Civil servants	Politicians	Service managers and	Researchers	Other external actors
van der Arend (2014)	1,2,8,11,24,25,31,32,36,37, 44,45,49	√		√			√		√	√			√	√							√	√		√				
von Lengerke et al. (2004)	57,58,59,62																√	√			√	√	√	√		√		√
Walter, Nutley and Davies (2005)	10,12,13,25,27,28,29,30,50			√	√		√	√						√							√	√	√	√		√	√	
Williamson et al. (2015)	20,25,36,39,40,42,44					√	√			√	√	√	√								√	√	√	√		√	√	√
Wye et al. (2015)	12,22,26,45,49,52				√		√						√	√	√								√	√		√		
Yost et al. (2014)	25,27,31,46						√	√	√				√										√	√				
Zardo and Collie (2014)	9,21,53			√		√									√						√			√				
Zardo and Collie (2015)	6,15,18		√		√	√															√			√				
Zardo, Collie and Livingstone (2015)	12,13,27,30,31,33,42,48,49				√			√	√			√		√								√		√				

Individual factors

Management of research integration

Organisational systems and infrastructure for research use

Institutional structures and rules for policymaking

Organisational characteristics

Policy level

Population
